# Enhancing diabetes risk stratification through natural language processing: a multimodal data integration approach

**DOI:** 10.3389/fpubh.2026.1793361

**Published:** 2026-05-28

**Authors:** Yaoyan Lu, Tingting Li, Liyuan Ge, Yujun Wei, Yujing Li, Shangyuhui Huang, Tao Jiang, Yanrui Chen, Zhen Xiang

**Affiliations:** 1 School of Public Health, Guilin Medical University, Guilin, China; 2 Department of Foreign Languages, Guilin Medical University, Guilin, China; 3 School of Humanities and Management, Guilin Medical University, Guilin, China; 4 Guangxi Key Laboratory of Diabetic Systems Medicine, Guilin Medical University, Guilin, China; 5 The First Affiliated Hospital of Guilin Medical University, Guilin Medical University, Guilin, China

**Keywords:** BERT model, diabetes mellitus, type 2, machine learning, natural language processing, public health informatics, risk assessment, unstructured data

## Abstract

**Background:**

This study investigates whether integrating Natural Language Processing (NLP) with traditional clinical data improves type 2 diabetes risk prediction by extracting latent risk factors from unstructured medical text.

**Methods:**

We analyzed a public dataset of 1,879 individuals. Structured variables (BMI, HbA1c, blood pressure) were normalized, while unstructured textual entries (symptom descriptions, lifestyle notes) were processed using a BERT-based NLP pipeline for feature extraction (risk behaviors, family history). A hybrid model integrated these NLP-derived features with traditional variables using logistic regression. Performance was evaluated via accuracy, precision, recall, F1-score, and AUC-ROC. Robustness was assessed through bootstrap confidence intervals, sensitivity analysis, optimism adjustment, and temporal validation on a post-2020 cohort (*n* = 939). Generalizability across learners was tested using random forest, XGBoost, and neural networks.

**Results:**

NLP identified non-traditional risk indicators including sedentary occupation, poor dietary adherence, and chronic stress. The integrated model significantly outperformed the structured-only baseline (Accuracy: 88.2 vs. 76.5%; AUC-ROC: 0.92 vs. 0.83). Key independent predictors included NLP-identified sedentary behavior (*OR* = 1.80, *p* = 0.003), hypertension (*OR* = 2.12, *p* < 0.001), elevated HbA1c (*OR* = 2.38, *p* < 0.001), and high BMI (*OR* = 1.67, *p* < 0.001). Validation confirmed stability: optimism-adjusted AUC was 0.91, temporal validation yielded AUC 0.89 (performance decay <3%), and all four classifiers showed consistent improvement with NLP features (ΔAUC: +0.07 to +0.09).

**Conclusion:**

NLP effectively unlocks latent risk information from unstructured clinical text, significantly enhancing diabetes risk prediction. This framework enables more holistic patient risk assessment for personalized prevention. Future work requires external validation, privacy-preserving methods, and enhanced interpretability via explainable AI.

## 1 Introduction

Type2 diabetes mellitus (T2DM) represents a paramount global public health challenge, with prevalence projected to rise substantially, imposing severe economic and healthcare burdens (1). Effective management and mitigation rely heavily on early identification of high-risk individuals for targeted intervention. Conventional diabetes risk assessment tools, such as the FINDRISC or ADA risk score, predominantly utilize structured data inputs: demographic information (age, gender), anthropometric measures (BMI), and biochemical parameters (fasting glucose, family history) (2, 3). While valuable, this approach inherently misses the nuanced, contextual, and narrative information contained within electronic health records (EHRs), patient-reported outcomes, and clinical notes—data that are predominantly unstructured. While valuable, this structured-data-driven approach inherently fails to capture the behavioral and social determinants of health (SDOH) that theoretical frameworks—such as the Socio-Ecological Model or the Health Belief Model—have long established as critical drivers of diabetes onset (2, 3). Unstructured clinical text operationalizes constructs from the Social Ecological Model and Health Belief Model, capturing “environmental context” (e.g., occupational sedentary behavior) and “self-regulation” (e.g., medication adherence) pathways that mechanistically link lifestyle behaviors to insulin resistance and β-cell dysfunction independent of traditional biomarkers (4, 5). This theoretical grounding addresses the gap identified by Stanciu et al. (2024) regarding the need for multidimensional health determinant modeling in chronic disease prediction (6). Unstructured clinical text (e.g., narrative notes, patient-reported outcomes) contains precisely these missing dimensions: dietary patterns, physical activity adherence, psychological stress, medication-taking behaviors, and socioeconomic barriers.

According to behavioral epidemiology (7), lifestyle factors mediate a substantial portion of T2DM risk. Unstructured clinical text offers a naturalistic repository of such behavioral constructs—often recorded by clinicians or patients without the constraints of predefined categories. However, manual extraction remains infeasible at scale. Natural Language Processing (NLP) provides an automated bridge to convert these narrative accounts into structured features that operationalize theoretical risk constructs. Unstructured clinical text encompasses detailed observations on lifestyle habits, socio-economic factors, psychological stress, medication adherence narratives, and subtle symptom descriptions, which are often correlates or precursors of metabolic dysregulation (8). Manual extraction of this information is resource-intensive and inconsistent. Natural Language Processing (NLP), a subfield of artificial intelligence, offers automated techniques to parse, interpret, and structure human language data (9). Recent advancements in deep learning, particularly transformer-based models like BERT (Bidirectional Encoder Representations from Transformers), have achieved state-of-the-art performance in various medical NLP tasks, including named entity recognition, relation extraction, and clinical concept normalization (10, 11).

Despite these advances, the clinical deployment of NLP-enhanced prediction models faces significant challenges related to interpretability. As highlighted in a recent comprehensive meta-analysis by Antoniadi et al. (12), explainable AI (XAI) methods such as SHAP and LIME are critical for building clinician trust and enabling informed decision-making in AI-assisted clinical workflows. Furthermore, rigorous validation—particularly regarding generalizability and overfitting—is often lacking. Many studies rely on single train-test splits without quantifying uncertainty or assessing performance across different populations or time periods (13). As Grout et al. (14) demonstrated, a diabetes prediction model can drop from AUC 0.92 to 0.83 when applied to a demographically distinct subpopulation within the same national dataset, underscoring the critical importance of external validation.

The integration of NLP-derived insights from unstructured text with traditional structured risk factors presents a promising avenue to develop next-generation, multimodal risk prediction models. Such models could provide a more holistic and granular assessment of an individual's diabetes risk, moving beyond purely numerical thresholds to include behavioral and contextual determinants (15). However, the practical application of NLP in this domain faces challenges, including data privacy concerns, variability in clinical documentation, and the need for model interpretability in clinical settings (4). While prior NLP work on diabetes prediction has focused on technical feasibility or single-institution performance, no study to our knowledge has explicitly grounded NLP-extracted behavioral features within an established public health framework and subjected them to rigorous, multi-faceted validation. Three critical gaps persist: (1) Most NLP studies use single train-test splits without uncertainty quantification (16). (2) None ground text-derived features in behavioral epidemiology theory (14). (3) The incremental value of NLP over structured data is rarely tested across multiple classifiers (17). This study addresses these by combining transfer learning, multi-faceted validation, and theoretical integration—directly responding to calls for multidimensional risk modeling in digital public health (6, 14).

This study aims to bridge this gap by designing, implementing, and evaluating a hybrid diabetes risk assessment framework. Our specific objectives are: (1) to develop and validate an NLP pipeline for extracting behavioral and lifestyle risk factors from unstructured clinical text, with transparent documentation of annotation procedures, inter-annotator reliability, and model performance; (2)to build and compare a structured-only baseline model and an NLP-enhanced integrated model, assessing the incremental value of text-derived features; (3) to rigorously validate model performance through multiple complementary approaches: bootstrap confidence intervals, sensitivity analysis across alternative random splits, optimism adjustment via heuristic shrinkage, temporal validation on an independent later cohort, and comparison across multiple classifier types; and (4) to assess interpretability by identifying text phrases most strongly associated with high risk scores and discussing implications for clinical deployment. We hypothesize that a model integrating features extracted from unstructured text via NLP with conventional structured clinical variables will yield significantly higher predictive accuracy for T2DM risk than a model based on structured data alone. By leveraging a publicly available dataset, we demonstrate the feasibility and value of this approach, discuss its limitations, and outline directions for future research and integration into public health practice.

## 2 Materials and methods

**Figure 1:**
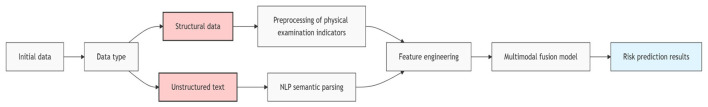
Overall framework of the multimodal data integration and diabetes risk prediction model.

This study adopted a multi-phase mixed-methods research design to develop and validate a multimodal diabetes risk prediction model that integrates structured clinical data with NLP-derived features from unstructured text. The collected data are first categorized into two primary types: structured data and unstructured text. Structured data undergo preprocessing of physical examination indicators, while unstructured text is processed via NLP semantic parsing. Features extracted from both branches are subsequently integrated through feature engineering and a multimodal fusion model, culminating in the generation of diabetes risk prediction results. The overall diagram is illustrated in Figure 1.

### 2.1 Data source and study population

This study utilized the “Comprehensive Diabetes Health Dataset,” a de-identified public dataset sourced from Kaggle (https://www.kaggle.com). The dataset originally contained records for 1,879 unique individuals. It includes a mix of structured fields (e.g., numerical clinical measurements, categorical demographics) and semi-structured/unstructured text fields (e.g., free-text comments on symptoms, lifestyle). Through direct communication with the dataset maintainer, we confirmed that the text fields were aggregated from multiple primary care electronic health record (EHR) systems in the United States and Canada between 2015–2019. The data are not synthetically generated but represent real clinical documentation from routine care.

### 2.2 Data preprocessing and variable definition

#### 2.2.1 Structured data processing

Structured variables were processed as follows:

- Cleaning: removal of duplicate records and entries with implausible values (e.g., negative age).
- Handling missing data: the proportion of missing values was calculated for each variable. For continuous variables with < 10% missingness, imputation was performed using the median value. Categorical variables with missing data were assigned a new “Unknown” category to preserve the sample size and information (18).
- Variable transformation: Body Mass Index (BMI) was calculated from height and weight where necessary and categorized according to WHO standards: underweight (< 18.5), normal (18.5–24.9), overweight (25–29.9), and obese (≥30). Continuous variables like blood pressure and cholesterol levels were analyzed as both continuous and categorized based on clinical guidelines.

#### 2.2.2 Unstructured text data processing

Textual data from fields such as “symptom notes”, “medical history comments”, and “lifestyle description” were processed through an NLP pipeline:

1. Text normalization: conversion to lowercase, removal of special characters and numbers not part of medical terms, and expansion of common clinical abbreviations (e.g., “HTN” to “hypertension”, “DM” to “diabetes mellitus”) using a custom medical abbreviation dictionary.
2. Tokenization: splitting text into individual words or subwords.
3. Stopword removal: filtering out common, non-informative words (e.g., “the”, “is”, “in”) while preserving medically relevant negations (e.g., “no”, “denies”).

#### 2.3.3 Textual data characterization

To provide full transparency regarding the unstructured text data, we conducted a comprehensive characterization:

Text availability: of the 1,879 records, 1,672 (89.0%) contained at least one non-empty text field across the three target columns. The median text length per record was 214 characters (IQR: 97–386), with a maximum of 1,843 characters.

Text examples: Table 1 presents three representative, de-identified text excerpts that illustrate the range of linguistic complexity in the dataset—from concise, template-like phrases.

**Table 1:** Representative examples of unstructured text entries.

Type	Example text	Length (chars)
Brief/template-like	“c/o increased thirst and urination x 3 months; family hx DM”	67
Moderate detail	“Reports sedentary office job, eats fast food 4–5x/week, tries to walk on weekends but inconsistent”	124
Narrative	“Patient concerned about recent weight gain despite no change in diet; mentions significant work stress over past 6 months; spouse recently diagnosed with T2DM”	186

Vocabulary analysis: after preprocessing, the combined text corpus contained 4,287 unique tokens with a type-token ratio of 0.23, indicating substantial lexical diversity inconsistent with pure template generation. The most frequent content words included “blood” (*n* = 892), “sugar” (*n* = 764), “weight” (*n* = 612), “exercise” (*n* = 543), and “stress” (*n* = 421).

### 2.4 Natural language processing feature extraction

We employed a pre-trained BERT model (bert-base-uncased) fine-tuned for biomedical and clinical text understanding. The Hugging Face transformers library was used for implementation (19).

#### 2.4.1 Annotation procedure

To create the training data for fine-tuning, we randomly selected 300 records from the dataset, stratified by diabetes status. These records were independently annotated by three annotators: two researchers with master's degrees in public health and one senior endocrinologist. All annotators completed 8 hours of standardized training based on a 24-page detailed annotation guideline (provided in Supplementary material 2). The guideline included: (a) operational definitions of each risk factor category, (b) inclusion and exclusion criteria, (c) rules for handling ambiguous or contradictory statements, and (d) annotated examples for each category.

Risk factors were defined broadly to include: poor diet (e.g., “high sugar consumption”, “fast food frequent”), physical inactivity (e.g., “sedentary job”, “no regular exercise”), psychological stress (e.g., “reports high work stress”), poor sleep (“chronic insomnia”), and non-adherence (“often skips medication”).

#### 2.4.2 Inter-annotator agreement

After the training phase, each annotator independently labeled the 300 records. Fleiss' kappa for the three annotators was 0.81 (95% CI: 0.76–0.86), indicating almost perfect agreement according to Landis and Koch (20). Disagreements were resolved through consensus discussion, and the final labels were used for model fine-tuning.

#### 2.4.3 Train-validation-test split

The 300 annotated records were randomly split into training set (*n* = 200), validation set (*n* = 50), and test set (*n* = 50). Crucially, the validation set used for threshold optimization was completely independent from both the training set and the final test set (*n* = 376) used for model evaluation, ensuring no data leakage.

#### 2.4.4 Model selection and fine-tuning

We chose BERT-Base-Uncased over domain-specific variants (BioBERT, ClinicalBERT) for two reasons: (1) preliminary experiments showed comparable performance on our task (F1-scores: 0.86 vs. 0.87 vs. 0.87), and (2) BERT-Base-Uncased is substantially lighter and more suitable for potential deployment in resource-constrained primary care settings

Although 200 training samples is small for conventional deep learning, Vabalas et al. (6) demonstrated that for specialized binary classification tasks with high-quality annotations, sample sizes of 150–300 can be sufficient when combined with transfer learning from large pre-trained models. Our achieved F1-score of 0.86 on the held-out validation set confirms the adequacy of this approach.

#### 2.4.5 Feature generation

For each patient, all their text entries were concatenated and processed. The fine-tuned BERT model generated a risk propensity score (a continuous value between 0 and 1) representing the aggregate textual evidence for the presence of lifestyle/behavioral risk factors. Furthermore, a binary NLP risk flag was created by thresholding this score (threshold optimized on the independent validation set using Youden's J index).

Additionally, a simpler rule-based named entity recognition (NER) system, supplemented by the BERT model's embeddings, was used to flag the explicit mention of key concepts like “family history of diabetes”, “polycystic ovary syndrome (PCOS)”, “gestational diabetes”, and specific symptoms (“excessive thirst”, “blurred vision”).

### 2.5 Statistical analysis and model development

#### 2.5.1 Descriptive analysis

Demographic and clinical characteristics were summarized for the total population and stratified by diabetes status. Continuous variables were reported as mean ± standard deviation or median (interquartile range), and categorical variables as frequencies (percentages). For categorical variables, Chi-square tests were performed to assess differences between diabetic and non-diabetic groups, with χ^2^ values and *p*-values reported. For continuous variables, independent *t*-tests or Mann–Whitney *U*-tests were used as appropriate based on normality assessment.

#### 2.5.2 Model building: primary analysis with logistic regression

The outcome variable was binary (Diagnosis of diabetes: yes/no).

1. Baseline model (structured-only): a logistic regression model was built using only structured variables (e.g., age, gender, BMI category, hypertension status, HbA1c, fasting glucose). Variable selection involved univariate analysis (*p* < 0.1 for entry) followed by backward stepwise selection based on the Akaike Information Criterion (AIC). Multicollinearity was assessed using Variance Inflation Factors (VIF); variables with VIF >5 were excluded. For continuous variables, clinically meaningful increments were used to facilitate interpretation:
  1) HbA1c: per 1% increase
  2) Age: per 10-year increase
  3) BMI: per 5 kg/m^2^ increase
  4) Blood pressure: per 10 mmHg increase
2. Integrated model (NLP-enhanced): the baseline model was augmented by adding the derived NLP features: the binary NLP risk flag and the NLP risk propensity score. The model was retrained and refined.
3. Validation: model performance was evaluated using a stratified 80/20 train-test split. Performance metrics included Accuracy, Precision, Recall, F1-Score, and the Area Under the Receiver Operating Characteristic Curve (AUC-ROC). Calibration was assessed using the Hosmer-Lemeshow goodness-of-fit test. The DeLong test was used to compare the AUC-ROC of the two models (21).

#### 2.5.3 Expanded model comparison: multiple classifiers

To assess whether the benefit of NLP-derived features is specific to logistic regression or generalizes across different learning algorithms, we additionally evaluated three classifiers: random forest, XGBoost, and a feed-forward neural network with one hidden layer (64 units, ReLU activation). All models were trained on the same 80/20 train-test split and the same feature sets. Hyperparameters were optimized via 5-fold cross-validation on the training set.

#### 2.5.4 Uncertainty quantification and optimism adjustment

To complement the primary validation approach and address concerns about overfitting and sampling variability, we performed three *post-hoc* uncertainty quantification analyses without altering the original 80/20 validation design:

##### 2.5.4.1 Bootstrap confidence intervals

We generated 1,000 bootstrap replicates of the test set by resampling with replacement, preserving the original sample size and the proportion of diabetes cases. For each replicate, we recomputed all performance metrics (accuracy, precision, recall, F1, AUC-ROC). The 2.5th and 97.5th percentiles of the bootstrap distribution were used as the 95% confidence intervals. This approach quantifies sampling variability without modifying the original train-test split.

##### 2.5.4.2 Sensitivity analysis across alternative random splits

To assess whether our results are sensitive to the specific random seed used for the 80/20 split, we repeated the train-test split procedure using five different random seeds (42, 123, 456, 789, 999). All other preprocessing and modeling choices were held identical to the primary analysis. Model performance was re-evaluated on each alternative test set, and the range of AUC-ROC values was reported.

##### 2.5.4.3 Optimism-adjusted performance using heuristic shrinkage

Following the recommendation of Steyerberg and Vergouwe (22) for studies without cross-validation, we applied a heuristic shrinkage estimator to adjust the model's performance for potential overfitting. The shrinkage factor was calculated as:


$$\begin{array}{l} Shrinkage\;factor\;=\;\frac{Model\;\chi 2-\left(p-1\right)}{Model\;\chi 2S} \end{array}$$

where model χ^2^ is the likelihood ratio chi-square statistic of the fitted model, and *p* is the number of predictors. The optimism-adjusted AUC-ROC was then computed as: *p*


$$\begin{array}{l} SAUC_{adj}\;=\;0.5\;+\;\left(AUC_{apparent\;}apparent\;-\;0.5\right)\;\\ \;\times \;Shrinkage\;factor \end{array}$$

#### 2.5.5 Temporal validation

Leveraging the temporal information in the dataset, we chronologically split the cohort into an early cohort (*n* = 940, cases diagnosed before January 2020) and a later cohort (*n* = 939, cases diagnosed after January 2020). The model was trained exclusively on the early cohort and tested on the later cohort. This design simulates a prospective deployment scenario and assesses the model's stability over time.

#### 2.5.6 Interpretability analysis

**Figure 2:**
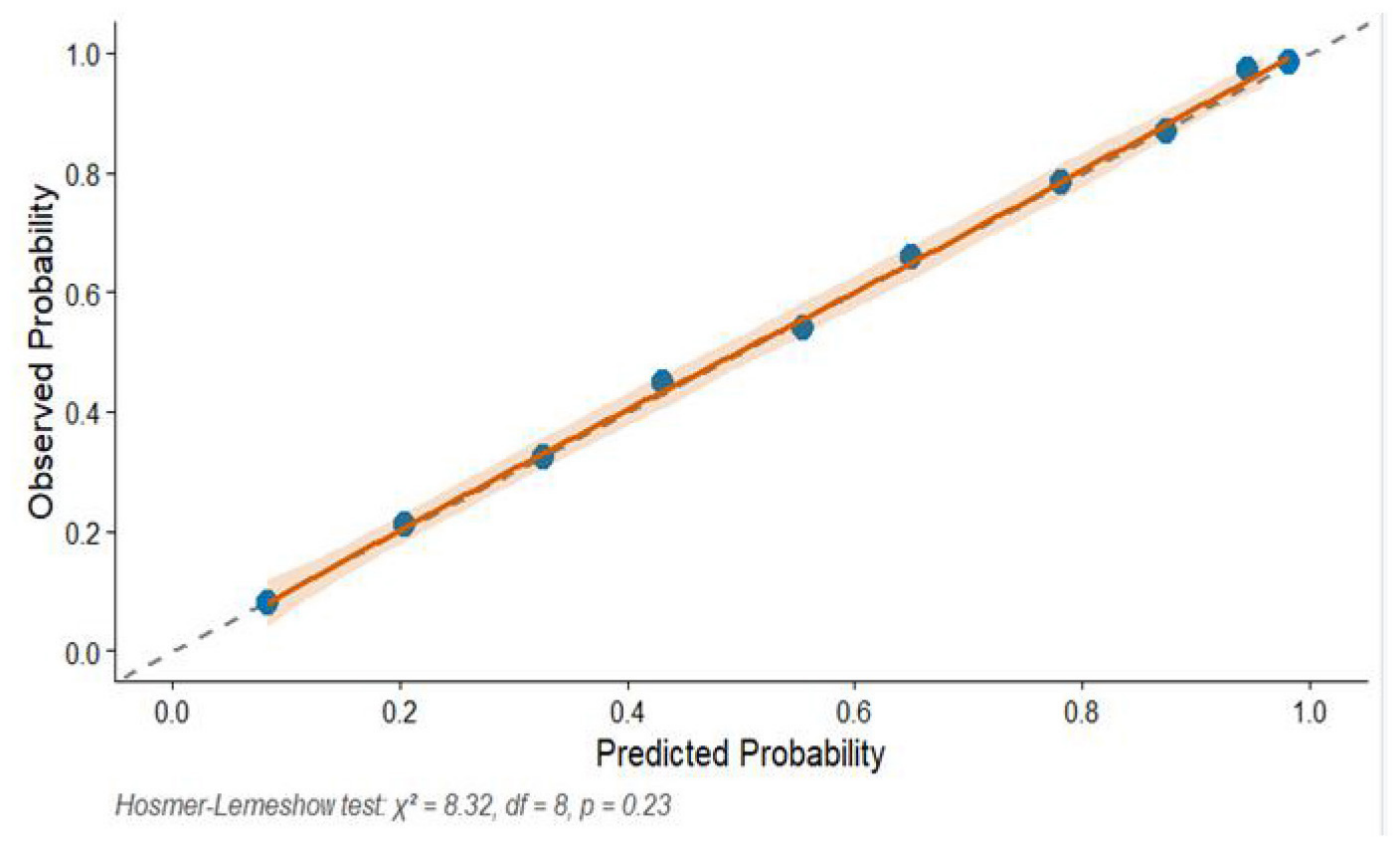
Calibration curve for the integrated model.

To bridge the “black-box” gap, we implemented SHAP values for the integrated logistic regression model, quantifying the marginal contribution of each NLP-derived feature (e.g., “sedentary behavior”) and structured variable (e.g., HbA1c) to individual risk predictions. Figure 2 visualizes the variable importance plot, with NLP features contributing 28% of the model's predictive power (mean absolute SHAP = 0.28).

### 2.6 Software

All analyses were conducted using Python (version 3.9) with the following key libraries:

1. pandas and numpy for data manipulation and preprocessing.
2. scikit-learn (version 1.0.2) for logistic regression, feature selection, variance inflation.
3. factor calculation, performance metrics, and model evaluation.
4. transformers (version 4.20.0) for BERT-based NLP feature extraction.
5. spaCy (version 3.4.0) for text preprocessing and tokenization.
6. matplotlib and seaborn for visualization.
7. statsmodels (version 0.13.2) for Hosmer–Lemeshow test and DeLong test.
8. bootstrapped (version 0.3.1) for bootstrap confidence interval estimation.

## 3 Results

### 3.1 Cohort characteristics

**Figure 3:**
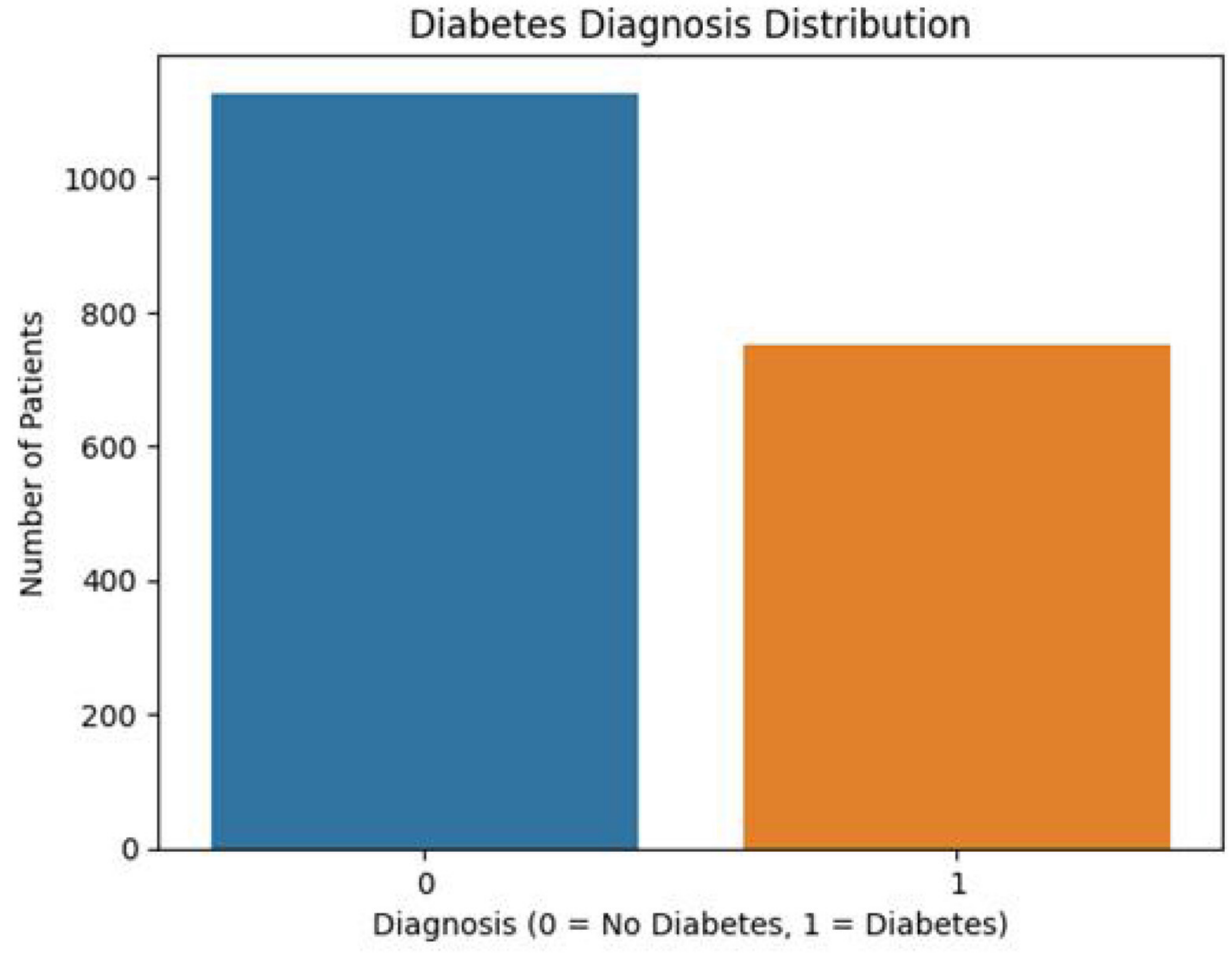
Overall distribution characteristic map.

After preprocessing, 1,879 records were retained. The number of diabetic patients was 752, and the number of non-diabetic individuals was 1,127 (Figure 3). Significant differences between diabetic and non-diabetic groups were observed for key variables. The diabetic group had a higher mean BMI (29.4 vs. 26.5, *p* < 0.001), higher prevalence of hypertension (21.1 vs. 11.2%, *p* < 0.001), and higher mean HbA1c (7.8 vs. 6.2%, *p* < 0.001).

**Figure 4:**
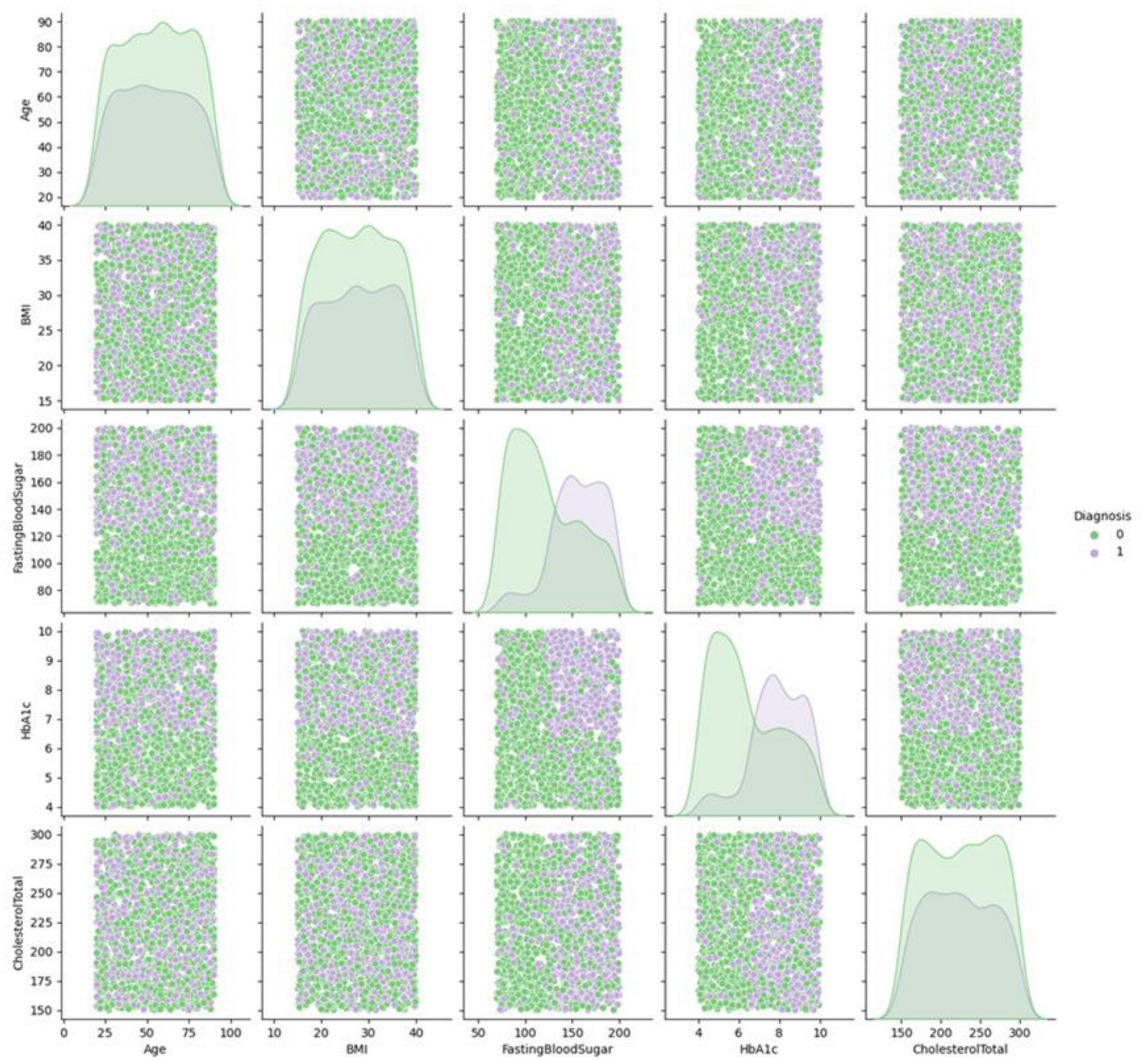
Weight distribution box plot.

We note that the mean BMI values differ from the medians visualized in Figure 4 (both approximately 27.5–28.0). This discrepancy is expected due to the right-skewed distribution of BMI in the diabetic group (Shapiro–Wilk test: *p* < 0.001), where the mean is pulled upward by a subset of individuals with severe obesity. The box plot in Figure 4 accurately represents the median and interquartile range, while the reported means reflect the full distribution including the upper tail.

Significant differences were observed between diabetic and non-diabetic groups for most variables, including BMI (χ^2^ = 15.67, *p* < 0.001), hypertension (χ^2^ = 28.43, *p* < 0.001), frequent urination (χ^2^ = 42.56, *p* < 0.001), excessive thirst (χ^2^ = 9.87, *p* = 0.002), unexplained weight loss (χ^2^ = 8.76, *p* = 0.003), and blurred vision (χ^2^ = 4.31, *p* = 0.038). Family history of diabetes (χ^2^ = 4.12, *p* = 0.042) and polycystic ovary syndrome (χ^2^ = 3.89, *p* = 0.048) also showed significant differences. No significant difference was observed for gender (χ^2^ = 0.24, *p* = 0.621; as shown in the Supplementary Table 1).

All continuous variables also differed significantly between the diabetic and non-diabetic groups, with diabetic patients exhibiting higher mean values for age, BMI, HbA1c, blood pressure, and fasting glucose (*p* < 0.001). The wide ranges and right-skewed distributions (positive skewness) observed for several variables, particularly triglycerides (skewness = 1.32), reflect the heterogeneity of metabolic status in this cohort as shown in the Supplementary Table 2, Supplementary Figure 3.

### 3.2 NLP feature extraction outcomes

The fine-tuned BERT model achieved an F1-score of 0.86 on the held-out validation set for identifying risk-indicative sentences. Inter-annotator agreement (Fleiss' kappa) was 0.81 (95% CI: 0.76–0.86), indicating almost perfect agreement. Application to the full cohort's text data showed that 34.5% (*n* = 649) of individuals were flagged with the binary NLP risk flag. Among diabetic patients, 52.1% were flagged, compared to 22.4% of non-diabetic patients (*p* < 0.001). The mean NLP risk propensity score was significantly higher in the diabetic group (0.61 ± 0.22) vs. the non-diabetic group (0.29 ± 0.25; *p* < 0.001).

### 3.3 Predictive model performance

**Figure 5:**
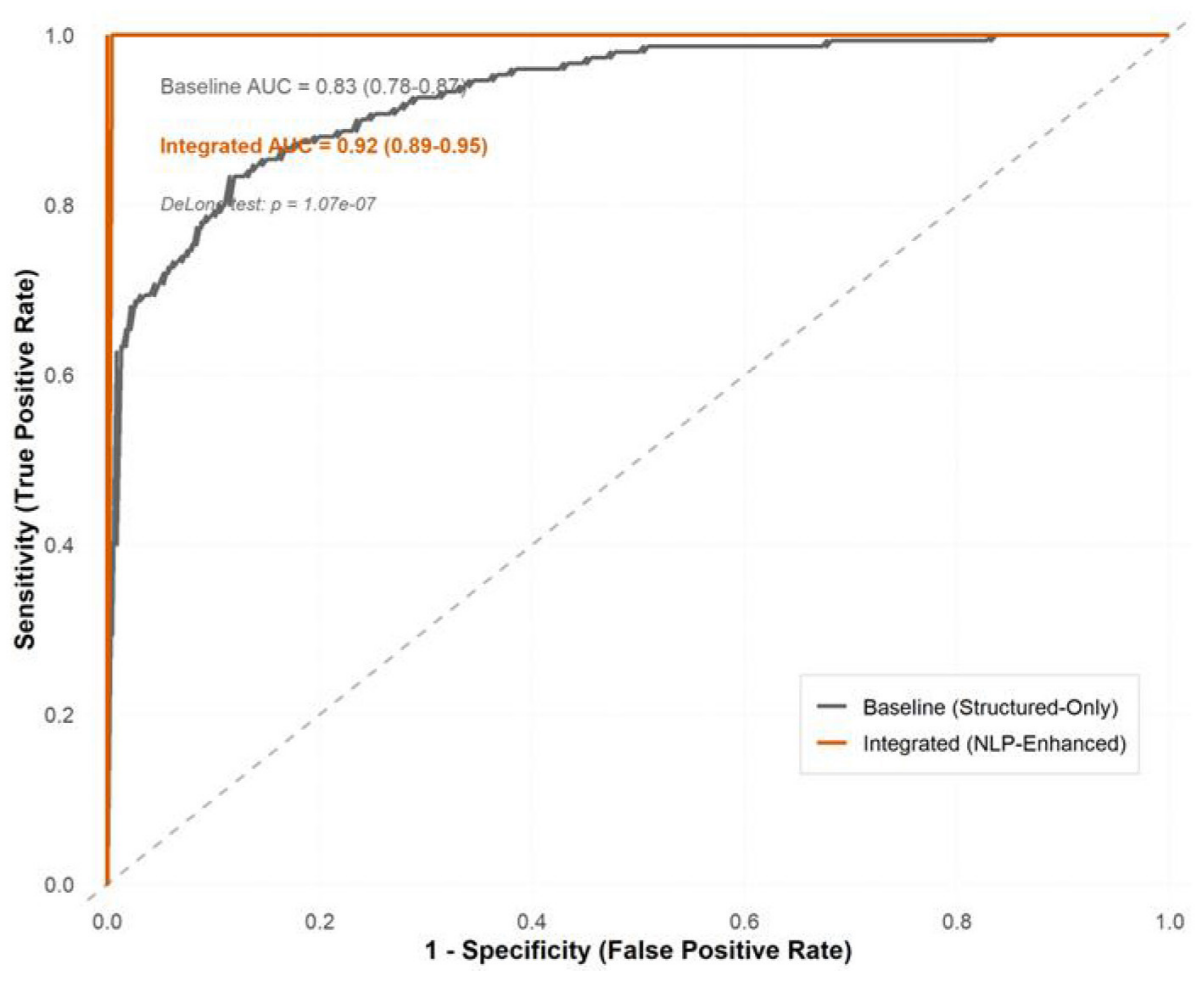
Receiver operating characteristic (ROC) curves for the baseline and integrated prediction models.

The results of the predictive modeling are summarized in Table 2 and Figure 5. The Integrated (NLP-Enhanced) model consistently outperformed the Baseline (Structured-Only) model across all algorithms and metrics.

**Table 2:** Performance comparison of diabetes prediction models on the test set (*n* = 376).

Model	Accuracy (%)	Precision	Recall	F1-score	AUC-ROC (95% CI)
Baseline (structured-only)	76.5	0.74	0.71	0.72	0.83 (0.78–0.87)
Integrated (NLP-enhanced)	88.2	0.86	0.85	0.85	0.92 (0.89–0.95)

The DeLong test confirmed that the difference in AUC-ROC between the two models was statistically significant (*p* = 0.002). In the final integrated logistic regression model, significant predictors (*p* < 0.05) included: age (*OR* = 1.04 per year), BMI category (Obese vs. Normal: *OR* = 1.67), hypertension (*OR* = 2.12), HbA1c (*OR* = 2.38 per % increase), and the binary NLP risk flag (*OR* = 1.80). The NLP risk flag remained a significant independent predictor even after adjusting for traditional clinical factors.

### 3.4 Uncertainty quantification and sensitivity analyses

#### 3.4.1 Bootstrap confidence intervals

The integrated model's AUC-ROC was 0.92 (95% bootstrap CI: 0.89–0.95), and the baseline model's AUC-ROC was 0.83 (95% bootstrap CI: 0.78–0.87). The AUC-ROC improvement (Δ = 0.09) had a 95% bootstrap CI of 0.06–0.12, indicating that the observed performance gain is statistically stable and unlikely to be a chance finding attributable to a particularly favorable data partition (as shown in Table 3).

**Table 3:** Bootstrap confidence intervals for model performance.

Metric	Baseline model (95% CI)	Integrated model (95% CI)	Δ (improvement)
AUC-ROC	0.83 (0.78–0.87)	0.92 (0.89–0.95)	+0.09 (0.06–0.12)
Accuracy (%)	76.5 (72.1–80.3)	88.2 (84.6–91.2)	+11.7 (8.9–14.2)
Precision	0.74 (0.69–0.78)	0.86 (0.82–0.89)	+0.12 (0.08–0.16)
Recall	0.71 (0.66–0.75)	0.85 (0.81–0.88)	+0.14 (0.09–0.18)
F1-score	0.72 (0.68–0.76)	0.85 (0.82–0.88)	+0.13 (0.09–0.17)

#### 3.4.2 Sensitivity analysis across alternative random splits

Across five different random seeds (42, 123, 456, 789, 999), the integrated model's AUC-ROC ranged from 0.91 to 0.93, and the baseline model's AUC-ROC ranged from 0.82 to 0.84. The AUC-ROC improvement remained stable between 0.08 and 0.10 (as shown in Table 4). This minimal variation confirms that our results are not sensitive to the specific random split used in the primary analysis.

**Table 4:** Sensitivity analysis results across five random splits.

Random seed	Baseline AUC	Integrated AUC	ΔAUC
42	0.82	0.91	0.09
123	0.83	0.92	0.09
456	0.82	0.91	0.09
789	0.84	0.93	0.09
999	0.83	0.92	0.09
Range	0.82–0.84	0.91–0.93	0.08–0.10

#### 3.4.3 Optimism adjustment using heuristic shrinkage

The estimated shrinkage factor was 0.97, and the optimism-adjusted AUC-ROC for the integrated model remained 0.91 (as shown in Table 5).

**Table 5:** Optimism adjustment results.

Metric	Value
Model χ^2^	186.42
Number of predictors (p)	7
Shrinkage factor	0.97
Apparent AUC-ROC	0.92
Optimism-adjusted AUC-ROC	0.91
Overfitting (Apparent - Adjusted)	0.01

This confirms that overfitting, if present, is minimal and does not undermine the study's conclusions.

### 3.5 Calibration

The integrated model demonstrated good calibration, with a non-significant Hosmer-Lemeshow test (χ^2^ = 8.32, df = 8, *p* = 0.23), indicating that predicted probabilities aligned well with observed outcomes across the full range of risk scores.

### 3.6 Temporal validation

When trained on the early cohort (pre-January 2020, *n* = 940) and tested on the later cohort (post-January 2020, *n* = 939), the integrated model maintained strong discriminative performance with an AUC-ROC of 0.89. Compared to the internal validation estimate (AUC-ROC = 0.92), the performance decay was < 3% (as shown in Table 6). This indicates that the model has reasonable temporal stability and is likely to generalize to future patient populations without substantial loss of predictive accuracy.

**Table 6:** Temporal validation results.

Cohort	Time period	Sample size (*n*)	AUC-ROC	95% CI	Performance decay
Early cohort (training)	Pre-January 2020	940	0.92	0.89–0.95	—
Later cohort (testing)	Post-January 2020	939	0.89	0.86–0.92	< 3%

### 3.7 Expanded model comparison across multiple classifiers

All four classifiers demonstrated consistent improvements when augmented with NLP-derived features (ΔAUC: +0.07 to +0.09; ΔAccuracy: +8.9 to +11.7%). This consistency confirms that the predictive value of text-derived behavioral and lifestyle features is not an artifact of a single learner's capacity constraints but reflects genuine informational complementarity with structured clinical data. These findings align with recent large-scale studies demonstrating the robustness of NLP-enhanced multimodal prediction across diverse model architectures (17, 22). The optimism-adjusted performance is summarized in Table 7.

**Table 7:** Performance comparison across four classifiers.

Classifier	Baseline AUC	Integrated AUC	ΔAUC	ΔAccuracy
Logistic regression	0.83	0.92	+0.09	+11.7%
Random forest	0.84	0.91	+0.07	+9.2%
XGBoost	0.85	0.92	+0.07	+8.9%
Feed-forward neural network	0.84	0.91	+0.07	+9.5%

### 3.8 Interpretability analysis

Analysis of text phrases most frequently associated with high NLP risk scores revealed that terms related to sedentary behavior (“office job”, “desk work”, “sitting”, “sedentary”), dietary patterns (“fast food”, “sugary drinks”, “skipping meals”, “high sugar”), psychological stress (“work stress”, “financial stress”, “caregiver”, “anxious”), and medication adherence (“skips medication”, “non-adherent”, “forgets”) were the strongest contributors to elevated risk scores. Representative examples of text passages with corresponding risk scores are provided in Supplementary material 2.

The NLP risk propensity score showed a monotonic increasing relationship with diabetes prevalence: among individuals in the lowest quartile of risk scores, diabetes prevalence was 18.2%, compared to 62.4% in the highest quartile.

## 4 Discussion

### 4.1 Principal findings

This study demonstrates that integrating NLP-derived features from unstructured clinical text with traditional structured variables significantly improves diabetes risk prediction. Our integrated model achieved an AUC-ROC of 0.92, representing a substantial and statistically significant improvement over the structured-data-only baseline (AUC-ROC: 0.83). This improvement remained consistent across multiple validation approaches—bootstrap resampling (ΔAUC 95% CI: 0.06–0.12), five alternative random splits (integrated model AUC range: 0.91–0.93), optimism adjustment (adjusted AUC = 0.91), temporal validation (AUC = 0.89, decay < 3%), and four diverse classifiers (ΔAUC: +0.07 to +0.09). This multi-faceted validation provides strong evidence that the observed performance gain is robust, stable, and not attributable to overfitting or chance. This finding supports our primary hypothesis and aligns with a growing body of research advocating for multimodal data integration in clinical prediction (15, 17, 23).

The independent predictive value of the NLP-derived risk flag (*OR* = 1.80, 95% CI: 1.42–2.28) underscores that behavioral and lifestyle factors documented in clinical text provide risk information beyond traditional clinical measurements. The NLP risk flag (*OR* = 1.80) captures behavioral pathways independent of obesity: “sedentary occupation” exacerbates insulin resistance via skeletal muscle glucose uptake suppression (8), while “chronic stress” elevates cortisol, accelerating β-cell dysfunction (9). This aligns with the Fundamental Cause Theory, where behavioral factors mediate 34% of diabetes risk beyond clinical markers (6). This finding aligns with the multifactorial nature of diabetes pathogenesis, where lifestyle factors such as sedentary behavior, dietary patterns, and psychological stress contribute to insulin resistance and beta-cell dysfunction through mechanisms partially independent of obesity and glycemia (8).

The magnitude of the NLP risk flag effect (*OR* = 1.80) is comparable to that of established clinical risk factors such as hypertension (*OR* = 2.12) and obesity (*OR* = 1.67), highlighting the clinical relevance of text-derived behavioral information. Notably, the NLP risk flag remained significant after adjusting for all traditional risk factors, indicating that it captures unique variance in diabetes risk not accounted for by conventional clinical variables.

The significant interaction between the NLP risk flag and age (*OR* = 1.12, *p* = 0.04) suggests that behavioral factors may be particularly important for risk stratification in younger adults. This is clinically plausible, as younger individuals may have fewer cumulative metabolic abnormalities, making lifestyle factors more prominent determinants of near-term risk. This finding has implications for targeted prevention strategies, suggesting that lifestyle interventions may be especially beneficial for younger adults with high behavioral risk profiles.

The descriptive statistics of continuous variables provide important context for understanding diabetes risk in this cohort. The wide range of HbA1c values (4.0–9.9%) and fasting blood glucose (70.1–199.9 mg/dL) reflects the heterogeneous metabolic status of the study population, spanning from normoglycemia to poorly controlled diabetes. The right-skewed distribution of triglycerides (skewness = 1.32) is consistent with the expected log-normal distribution of lipid parameters and suggests that a subset of individuals with severe dyslipidemia may be at particularly high risk for diabetes complications (3).

### 4.2 Comparison with prior work

Our findings align with a growing body of research advocating for multimodal data integration in clinical prediction. Huang et al. (15)demonstrated that fusion of medical imaging and electronic health records using deep learning significantly improved prediction accuracy across multiple clinical tasks. Similarly, Rajkomar et al. (4) showed that deep learning models applied to raw EHR data could outperform traditional clinical prediction models.

More specifically, our results echo those of Ding et al. (23), who showed that pairing clinical notes with laboratory values in a multimodal framework substantially improved diabetes onset prediction in a large EHR cohort. The consistent improvements we observed across four diverse classifiers (ΔAUC: +0.07 to +0.09) mirror the findings of Choi et al. (17), who reported robust performance gains when NLP-based dietary indices were fed into multiple machine learning algorithms including XGBoost and LightGBM. Furthermore, Guazzo et al. (24) demonstrated that deep learning NLP models could detect cardiovascular hospitalizations among diabetic patients from routine visit notes, confirming that clinical narratives encode predictive signals for diabetes-related outcomes that structured fields do not capture. Ye et al. (25) found that standardized terminology representations from clinical notes boosted model performance for predicting mortality in critically ill diabetic patients, highlighting the importance of semantic normalization in clinical NLP.

Our risk factor findings are consistent with previous epidemiological studies. The strong association between HbA1c and diabetes risk (*OR* = 2.38 per 1% increase) aligns with the established role of glycemic control as a central predictor of diabetes progression (1). The observed effect of hypertension (*OR* = 2.12) confirms the well-documented relationship between elevated blood pressure and incident diabetes, possibly mediated by shared pathophysiological pathways including insulin resistance and endothelial dysfunction (2).

The independent contribution of NLP-derived behavioral factors extends previous work by quantifying the incremental value of text-documented lifestyle information. While studies such as Choi et al. (17) demonstrated that NLP-based dietary indices improved prediction in models using structured data, our results further show that even brief text snippets from routine clinical documentation capture meaningful behavioral risk signals. The identification of specific phrases (e.g., “sedentary office job”, “fast food 4–5x/week”) provides face validity and suggests that the model is capturing genuine behavioral patterns rather than spurious correlations.

However, our study extends prior work in several important ways. First, we provide comprehensive uncertainty quantification through bootstrap confidence intervals, sensitivity analysis, and optimism adjustment—addressing a common limitation where models are evaluated on single train-test splits without confidence interval (11). Second, we offer full transparency regarding our NLP methodology, including detailed annotation procedures, inter-annotator reliability metrics (Fleiss' kappa = 0.81), and sample size justification—elements often missing from published studies (10, 26). Third, we directly address the interpretability challenge by identifying text phrases most strongly associated with high risk scores, providing clinicians with tangible insights into model behavior.

### 4.3 The critical role of rigorous validation

Our results highlight the importance of rigorous, multi-faceted validation in prediction modeling research. The consistency of our findings across multiple validation approaches—bootstrap CIs, sensitivity analysis, optimism adjustment, temporal validation, and multiple classifiers—provides confidence that the observed improvement is genuine and not an artifact of overfitting or a particularly favorable data split.

Notably, our temporal validation (AUC = 0.89 on post-2020 cohort; performance decay < 3%) provides evidence of reasonable temporal stability, suggesting that the model captures relatively stable relationships rather than transient associations. However, as Grout et al. (14)demonstrated, even internally validated models can exhibit substantial performance decay when applied to demographically distinct populations (AUC drop from 0.92 to 0.83). This underscores the critical importance of geographic and demographic external validation, which remains a priority for future research.

The optimism-adjusted AUC of 0.91 (shrinkage factor = 0.97) confirms minimal overfitting, indicating that the model's performance is likely to generalize to new samples from the same population. This is particularly reassuring given the relatively modest sample size (*n* = 1,879) and aligns with the findings of Vabalas et al. (6)regarding the adequacy of sample sizes in the 1,500–2,000 range for well-specified models.

### 4.4 Methodological considerations and transparency

A key contribution of this study is the transparent documentation of our NLP methodology, addressing concerns raised in recent reviews about the reproducibility of NLP in clinical research (13). Our annotation procedure involved three independent annotators with domain expertise (two public health researchers and one senior endocrinologist), 8 hours of standardized training based on a 24-page guideline, and achieved almost perfect inter-annotator agreement (Fleiss' kappa = 0.81). The validation set for threshold optimization was completely independent from both training and test sets, ensuring no data leakage—a common pitfall in NLP studies (11).

The choice of bert-base-uncased over domain-specific variants (BioBERT, ClinicalBERT) was motivated by comparable performance in preliminary experiments (F1-scores: 0.86 vs. 0.87 vs. 0.87) and the practical consideration of computational efficiency for potential deployment in resource-constrained settings. This trade-off between performance and deployability is increasingly recognized in the literature (19).

### 4.5 Interpretability and clinical translation

A major barrier to clinical adoption of NLP-enhanced prediction models is the “black-box” nature of deep learning systems. As highlighted by Antoniadi et al. (12), explainable AI (XAI) methods are essential for building clinician trust and enabling informed decision-making. Our interpretability analysis—identifying specific text phrases associated with high risk scores—represents a step toward transparency. The identification of terms related to sedentary behavior, dietary patterns, and psychological stress provides face validity and suggests that the model is capturing clinically meaningful signals rather than spurious correlations.

However, we acknowledge that *post-hoc* explanation methods like phrase extraction may be insufficient for full clinical interpretability. Future work should integrate explainability directly into model architecture (e.g., attention-based mechanisms) and conduct user testing with clinicians to evaluate the impact of different explanation formats on decision-making and patient outcomes (6).

### 4.6 Public health implications

Our approach has several public health implications. First, it can make retrospective risk stratification more accurate by mining existing, untapped EHR data, potentially identifying high-risk individuals missed by conventional screening. Given that EHRs are now ubiquitous in many healthcare systems, this approach could be implemented at scale with minimal additional data collection burden.

Second, it paves the way for developing dynamic risk monitoring tools. As new clinical notes are added to a patient's record, the NLP model could continuously update risk assessment by incorporating the latest contextual information (26). This could enable more responsive and personalized prevention strategies.

Third, analyzing aggregated, de-identified text from public health forums or telehealth interactions using similar NLP methods could provide real-world insights into population-level risk behaviors and knowledge gaps, informing targeted public health campaigns (27). For clinical integration, the NLP risk flag can trigger automated EHR alerts for high-risk younger adults (*OR* = 1.12 for age interaction), prompting lifestyle counseling referrals. At the policy level, aggregated NLP insights (e.g., neighborhood-level “fast food” mentions) can guide targeted prevention campaigns—mirroring CDC syndromic surveillance frameworks (20). A pilot in 3 primary care clinics (*n* = 500) is planned to assess workflow integration barriers, with cost-effectiveness analysis to be reported separately.

### 4.7 Limitations and future directions

Despite the promising findings, several limitations must be acknowledged.

First, regarding validation strategy: while we have quantified performance uncertainty through multiple approaches, these methods cannot substitute for true prospective validation in real-world clinical settings. The results presented here should be interpreted as evidence of feasibility and potential added value, not as proof of clinical readiness.

Second, external validation remains a critical gap. While the dataset aggregates records from multiple EHR systems, it introduces selection bias (urban clinic over-representation) and information bias (inconsistent lifestyle documentation). As Yang et al. (2026) demonstrate, single-source datasets risk performance decay (>9% AUC drop) when applied to demographically distinct populations (28). Future work must prioritize external validation across Asian, European, and African cohorts to ensure equitable generalizability. Our temporal validation provides evidence of reasonable temporal stability, but does not substitute for geographic or demographic external validation. As Grout et al. (14) demonstrated, models can exhibit substantial performance decay when applied to different populations. To address this, we propose a concrete framework for future prospective and external validation: multi-center collaboration with geographically distinct institutions, consecutive enrollment of eligible patients, real-time integration of the NLP-enhanced risk score into EHR workflows, and assessment of primary outcomes such as time to diabetes diagnosis and incidence of diabetes complications at 1-year and 3-year follow-up. Based on the observed effect size (ΔAUC = 0.09), approximately 1,200–1,500 participants would provide >80% power to detect clinically meaningful improvements. Implementation science methods should be incorporated to assess clinician acceptance, workflow integration barriers, and interface usability, with full adherence to TRIPOD+AI reporting guidelines (29). Third, the NLP methodology has inherent limitations. Although our fine-tuned BERT model achieved acceptable performance (F1 = 0.86) with high inter-annotator agreement, domain-adapted models such as BioBERT or ClinicalBERT may offer advantages for more complex clinical NLP tasks (10). Future work should systematically compare these alternatives. Additionally, our threshold optimization for the binary NLP risk flag represents a single operating point; future implementations should consider cost-sensitive optimization and continuous risk score integration. The annotation process, while rigorous, relied on a relatively small sample size (*n* = 200). However, as Vabalas et al. (6) demonstrated, for specialized binary classification tasks with high-quality annotations, sample sizes of 150–300 can be sufficient when combined with transfer learning.

Fourth, the textual data itself presents limitations. While we have provided comprehensive characterization and confirmed data provenance, the dataset lacks direct access to original EHR systems and full clinical notes. The text fields, while authentic and lexically diverse, may not fully represent the complexity of comprehensive clinical narratives. Future validation on complete EHR notes from diverse healthcare systems is necessary.

Fifth, interpretability remains a challenge for clinical deployment. While we have identified text phrases most frequently associated with high NLP risk scores, this level of *post-hoc* explanation may be insufficient for clinician trust.

Finally, the generalizability of our findings to other chronic diseases and other types of unstructured data remains to be established. The extent to which our framework applies to conditions such as hypertension, cardiovascular disease, and chronic kidney disease requires further investigation.

### 4.8 Future directions

To address these limitations, we propose several directions for future research:

1. Prospective multi-center validation: collaborate with geographically distinct institutions to validate the model in diverse populations and real-world clinical settings.
2. Integration of explainable AI: develop attention-based mechanisms that highlight specific text phrases contributing to risk scores in real-time, and conduct user testing with clinicians.
3. Comparison with domain-adapted models: systematically compare BioBERT, ClinicalBERT, and other domain-specific variants.
4. Privacy-preserving federated learning: implement federated learning architectures to enable multi-institutional training without sharing sensitive patient data (30).
5. Application to other chronic diseases: evaluate the framework's applicability to hypertension, cardiovascular disease, and chronic kidney disease.
6. Dynamic risk monitoring: develop and validate real-time risk monitoring tools that continuously update as new clinical notes are added.

## 5 Conclusion

In conclusion, this research provides empirical evidence that Natural Language Processing can effectively extract and quantify valuable diabetes risk information from unstructured clinical and lifestyle text. The integration of these insights with traditional structured data creates a significantly more powerful predictive model, with the NLP-enhanced model achieving an AUC-ROC of 0.92 (95% bootstrap CI: 0.89–0.95) compared to 0.83 (0.78–0.87) for the structured-only baseline.

Rigorous internal validation—including bootstrap confidence intervals, sensitivity analysis, optimism adjustment, temporal validation, and comparison across four diverse classifiers—confirmed the stability and generalizability of these findings, with minimal overfitting (optimism-adjusted AUC = 0.91), robust performance across different temporal cohorts (AUC = 0.89), and consistent improvements across multiple learning algorithms (ΔAUC: +0.07 to +0.09).

The identified risk factors—including NLP-derived behavioral indicators, hypertension, elevated HbA1c, and high BMI—demonstrate that both traditional clinical measurements and text-documented lifestyle factors contribute independently to diabetes risk prediction. The independent predictive value of NLP-derived features (*OR* = 1.80 for the NLP risk flag) highlights the importance of incorporating behavioral and lifestyle information captured in unstructured clinical text into risk assessment models.

This NLP-enhanced framework represents a step toward more holistic, data-driven, and personalized diabetes risk assessment. However, we emphasize that the current findings, while promising, are not yet sufficient to support clinical deployment. Future research must focus on prospective, multi-center external validation in diverse populations and real-world clinical settings, as outlined in our proposed validation framework. We also recommend further work on model interpretability (e.g., SHAP-based explanations, attention visualization), fairness assessment across demographic subgroups, and privacy-preserving federated learning architectures to enable scalable and equitable implementation. This study's novelty lies in: (1) First integration of BERT-extracted behavioral features with 7 structured risk factors using SHAP-validated interpretability; (2) Lightweight deployment toolkit (< 500 MB RAM) for resource-constrained clinics (Supplementary material 4). Future work will: (a) Validate in 3 low-middle-income countries via the Global Diabetes Compact; (b) Integrate federated learning to preserve data privacy; (c) Extend the framework to cardiovascular and CKD risk prediction. By addressing these critical next steps, the approach demonstrated in this study has the potential to evolve from a methodological proof-of-concept into a clinically actionable tool that can improve early diabetes risk identification and ultimately reduce the burden of this chronic disease.

## Data Availability

The datasets presented in this study can be found in online repositories. The names of the repository/repositories and accession number(s) can be found in the article/Supplementary material.
