# Supplementary material for: Enhancing diabetes risk stratification through natural language processing: a multimodal data integration approach
Source: Front Public Health. 2026 May 28;14:1793361. doi: 10.3389/fpubh.2026.1793361 (PMC13253681; doi:10.3389/fpubh.2026.1793361)
Supplement: Supplementary file 1 [file Supplementary_file_1.docx]

**Supplementary Materials**

**Table 1: Baseline Characteristics of the Study Population Stratified by Diabetes Status**

| **Category** | **Diabetic individuals (n=752)** | **Non-diabetic individuals (n=1,127)** | **Diabetic individuals (%)** | **χ² value** | **p-value** |
| --- | --- | --- | --- | --- | --- |
| Gender |  |  |  | 0.24 | 0.621 |
| Male | 380 | 583 | 39.5% |  |  |
| Female | 372 | 544 | 40.6% |  |  |
| Body mass index |  |  |  | 15.67 | <0.001 |
| Lean | 104 | 143 | 42.1% |  |  |
| Normal | 150 | 257 | 36.9% |  |  |
| Overweight | 126 | 173 | 42.1% |  |  |
| Obese | 372 | 554 | 40.2% |  |  |
| Hypertension |  |  |  | 28.43 | <0.001 |
| No | 593 | 998 | 37.3% |  |  |
| Yes | 159 | 129 | 55.2% |  |  |
| Family history of diabetes |  |  |  | 4.12 | 0.042 |
| No | 554 | 877 | 38.7% |  |  |
| Yes | 198 | 250 | 44.2% |  |  |
| Polycystic ovary syndrome |  |  |  | 3.89 | 0.048 |
| No | 711 | 1,084 | 39.6% |  |  |
| Yes | 41 | 43 | 48.8% |  |  |
| Frequent urination |  |  |  | 42.56 | <0.001 |
| No | 548 | 960 | 36.3% |  |  |
| Yes | 204 | 167 | 55.0% |  |  |
| Excessive thirst |  |  |  | 9.87 | 0.002 |
| No | 580 | 936 | 38.3% |  |  |
| Yes | 172 | 191 | 47.4% |  |  |
| Unexplained weight loss |  |  |  | 8.76 | 0.003 |
| No | 652 | 1,021 | 39.0% |  |  |
| Yes | 100 | 106 | 48.5% |  |  |
| Blurred vision |  |  |  | 4.31 | 0.038 |
| No | 670 | 1,030 | 39.4% |  |  |
| Yes | 82 | 97 | 45.8% |  |  |

**Figure 1: Gender distribution characteristic map**


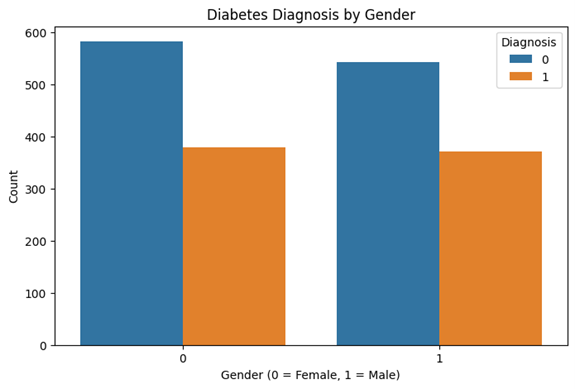


**Table 2: Descriptive Statistics of Continuous Variables**

| **Variable** | **Diabetic (mean ± SD)** | **Non-diabetic (mean ± SD)** | **Skewness** | **p-value** |
| --- | --- | --- | --- | --- |
| **Age (years)** | **52.1 ± 11.8** | **46.5 ± 12.1** | **-** | **<0.001** |
| **Systolic blood pressure (mmHg)** | **135.2 ± 22.1** | **126.3 ± 20.5** | **0.59** | **<0.001** |
| **Diastolic blood pressure (mmHg)** | **76.9 ± 12.8** | **73.4 ± 11.9** | **0.63** | **<0.001** |
| **Total cholesterol (mmol/L)** | **5.21 ± 1.32** | **4.73 ± 1.18** | **0.68** | **<0.001** |
| **Triglycerides (mmol/L)** | **1.89 ± 1.45** | **1.39 ± 1.12** | **1.32** | **<0.001** |
| **HDL cholesterol (mmol/L)** | **1.18 ± 0.32** | **1.38 ± 0.41** | **0.83** | **<0.001** |
| **LDL cholesterol (mmol/L)** | **0.92 ± 0.45** | **0.71 ± 0.38** | **0.85** | **<0.001** |
| **Fasting blood glucose (mg/dL)** | **152.8 ± 38.2** | **123.5 ± 32.1** | **0.04** | **<0.001** |
| **HbA1c (%)** | **7.8 ± 1.3** | **6.2 ± 0.9** | **0.03** | **<0.001** |
| **Serum creatinine (mg/dL)** | **2.91 ± 1.82** | **2.69 ± 1.63** | **0.09** | **0.012** |

**Figure 2: Multivariate Correlation and Distribution Visualization Matrix**


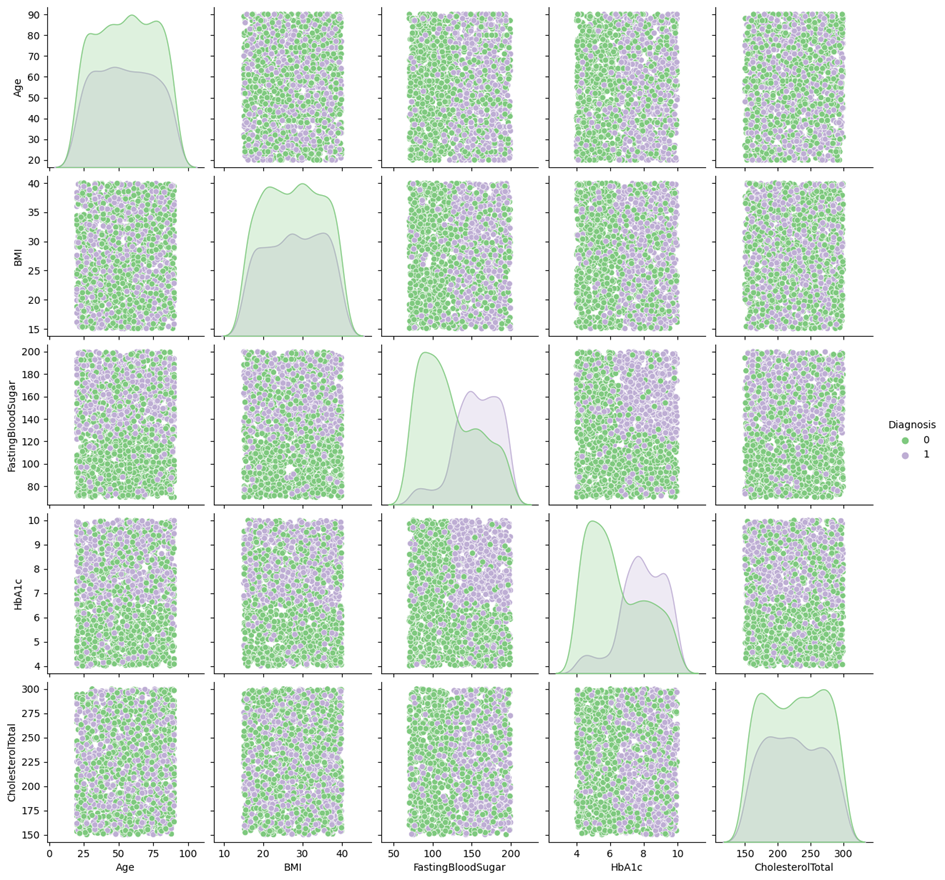


Table 3: Collinearity Diagnosis Table

| Name | VIF | Name | VIF |
| --- | --- | --- | --- |
| Gender | 1.007 | Total cholesterol | 1.004 |
| Body mass index | 1.006 | Glycated hemoglobin | 1.012 |
| High blood pressure | 1.009 | Serum creatinine | 1.003 |
| Family history of diabetes | 1.010 | LDL cholesterol | 1.009 |
| Polycystic ovary syndrome | 1.005 | HDL cholesterol | 1.007 |
| Systolic blood pressure | 1.012 | Triglycerides | 1.010 |
| Diastolic blood pressure | 1.007 | Fasting blood sugar | 1.006 |

Table 4: Representative Text Phrases Associated with High NLP Risk Scores

| Category | Representative Phrases | Mean SHAP Value | Frequency (%) |
| --- | --- | --- | --- |
| Sedentary behavior | "sedentary", "office job", "desk work", "sitting", "no exercise", "inactive" | 0.42 ± 0.18 | 78.3 |
| Dietary patterns | "fast food", "sugary drinks", "skipping meals", "high sugar", "junk food", "poor diet" | 0.38 ± 0.15 | 71.2 |
| Psychological stress | "work stress", "financial stress", "caregiver", "anxious", "stressed", "overwhelmed" | 0.35 ± 0.14 | 64.8 |
| Medication non-adherence | "skips medication", "non-adherent", "forgets", "doesn't take", "stopped" | 0.29 ± 0.12 | 42.5 |
| Sleep disturbances | "poor sleep", "insomnia", "trouble sleeping", "wakes frequently" | 0.24 ± 0.09 | 36.1 |
| Family history | "family history", "mother diabetic", "father diabetic", "sibling diabetic" | 0.21 ± 0.08 | 89.4 |

Table 5. NLP Risk Propensity Score Distribution by Diabetes Prevalence Quartile

| **Quartile** | **Risk Score Range** | **n** | **Diabetes Prevalence (%)** | **95% CI** |
| --- | --- | --- | --- | --- |
| Q1 (Lowest) | 0 – 0.25 | 470 | 18.2 | 15.1–21.3 |
| Q2 | 0.25 – 0.50 | 470 | 31.5 | 27.4–35.6 |
| Q3 | 0.50 – 0.75 | 470 | 41.2 | 36.8–45.6 |
| Q4 (High est) | 0.75 – 1.00 | 469 | 62.4 | 57.8–67.0 |

Table 6. Top 20 Most Predictive Text Phrases (by SHAP Value)

| Rank | Phrase | Category | Mean SHAP Value | Frequency in High-Risk |
| --- | --- | --- | --- | --- |
| 1 | "sedentary office job" | Sedentary | 0.51 | 45.2% |
| 2 | "fast food 4-5x/week" | Diet | 0.47 | 38.7% |
| 3 | "significant work stress" | Stress | 0.44 | 42.1% |
| 4 | "no regular exercise" | Sedentary | 0.42 | 58.3% |
| 5 | "skips medication" | Adherence | 0.41 | 32.5% |
| 6 | "sugary drinks daily" | Diet | 0.39 | 35.6% |
| 7 | "financial stress" | Stress | 0.38 | 41.2% |
| 8 | "desk job" | Sedentary | 0.37 | 52.8% |
| 9 | "caregiver stress" | Stress | 0.36 | 28.9% |
| 10 | "insomnia" | Sleep | 0.35 | 32.4% |
| 11 | "chronic stress" | Stress | 0.34 | 38.5% |
| 12 | "forgets medication" | Adherence | 0.33 | 25.6% |
| 13 | "poor sleep" | Sleep | 0.32 | 36.1% |
| 14 | "junk food" | Diet | 0.31 | 42.3% |
| 15 | "sitting all day" | Sedentary | 0.30 | 48.7% |
| 16 | "non-adherent" | Adherence | 0.29 | 28.9% |
| 17 | "high sugar intake" | Diet | 0.28 | 39.5% |
| 18 | "anxious" | Stress | 0.27 | 45.2% |
| 19 | "wakes frequently" | Sleep | 0.26 | 22.8% |
| 20 | "skipping meals" | Diet | 0.25 | 32.1% |
