# Supplementary material for: Enhancing diabetes risk stratification through natural language processing: a multimodal data integration approach
Source: Front Public Health. 2026 May 28;14:1793361. doi: 10.3389/fpubh.2026.1793361 (PMC13253681; doi:10.3389/fpubh.2026.1793361)
Supplement: Supplementary file 2 [file Supplementary_file_2.pdf]

Supplementary Material 2: Comprehensive Annotation Guideline for NLP Feature Extraction

Version 2.1 | Last Updated: April 2025

Associated Manuscript: *Enhancing Diabetes Risk Stratification through Natural Language Processing: A Multimodal Data Integration Approach*

1. Introduction & Purpose

1.1 Objective

This document provides the exhaustive operational guidelines used for annotating unstructured clinical text within the "Comprehensive Diabetes Health Dataset." The primary goal is to standardize the identification and extraction of behavioral, lifestyle, and socio-environmental risk factors for Type 2 Diabetes Mellitus (T2DM) from free-text clinical notes, symptom descriptions, and lifestyle narratives.

1.2 Scope

These guidelines govern the annotation of three specific text fields: Symptom Description, Lifestyle Notes, and Provider Comments. They apply to the 300 randomly sampled records used for fine-tuning the BERT model.

2. Theoretical Framework & Definitions

The annotation schema is grounded in the **Social Ecological Model** and **Health Belief Model**, targeting constructs that operate independently of traditional biomarkers.

Construct	Definition	Clinical Relevance to T2DM
**Behavioral Risk**	Observable actions impacting metabolic health.	Directly linked to insulin resistance and β-cell dysfunction.
**Environmental Context**	External conditions shaping behavior.	Influences opportunity for healthy choices (e.g., food desert, sedentary job).

Construct	Definition	Clinical Relevance to T2DM
**Self-Regulation**	Adherence to medical advice and self-management.	Predicts glycemic control and complication risk.

3. Annotation Team & Training Protocol

3.1 Team Composition

- **Annotator 1 & 2:** PhD candidates in Public Health (Epidemiology specialization). Trained in clinical terminology and risk factor taxonomy.
- **Annotator 3:** Senior Endocrinologist (15+ years clinical experience). Provided clinical validation and adjudication.

3.2 Training Curriculum (8 Hours)

1. **Lecture (2 hrs):** Overview of T2DM pathophysiology, risk factors, and the role of NLP in behavioral epidemiology.
2. **Guideline Walkthrough (2 hrs):** Detailed review of this document, focusing on edge cases and negation handling.
3. **Calibration Exercise (3 hrs):** Independent annotation of 50 pilot records not included in the final training set.
4. **Consensus Meeting (1 hr):** Resolution of discrepancies in the pilot set to establish a unified mental model.

4. Detailed Risk Factor Taxonomy

Each variable must be coded as **Present (1)**, **Absent (0)**, or **Uncertain/Missing (?)**.

4.1 Dietary Patterns (Poor Diet)

Targets the "Dietary Adherence" pathway.

- **High Sugar Consumption:** Explicit mention of frequent intake of sugary beverages, candies, desserts, or high-glycemic foods.
  - *Include:*"drinks soda daily", "eats sweets after every meal", "prefers juice

over water".
    - *Exclude:*"eats fruit", "uses artificial sweeteners".
- **Processed/Fast Food Reliance:** Dependence on convenience foods, takeout, or fried foods (>3 times/week).
    - *Include:*"fast food 4-5x/week", "relies on frozen dinners", "drive-thru daily".
    - *Exclude:*"home-cooked meals", "meal preps on Sundays".
- **Irregular Meal Patterns:** Skipping meals, binge eating, or erratic eating schedules.
    - *Include:*"skips breakfast", "binges at night", "only eats one meal a day".
    - *Exclude:*"intermittent fasting (planned)".

4.2 Physical Activity (Physical Inactivity)

Targets the "Skeletal Muscle Glucose Uptake" pathway.

- **Sedentary Occupation:** Job primarily involving sitting for >6 hours/day.
    - *Include:*"office job", "desk work", "truck driver", "call center agent".
    - *Exclude:*"construction worker", "nurse on feet all day".
- **Leisure Time Inactivity:** Absence of planned exercise or recreational movement (<150 mins/week moderate intensity).
    - *Include:*"no regular exercise", "watches TV all weekend", "hates gym".
    - *Exclude:*"walks dog daily", "plays sports weekly", "gardens".

4.3 Psychological & Sleep Factors

Targets the "Cortisol-Mediated Beta-Cell Dysfunction" pathway.

- **Chronic Stress:** Persistent psychological strain from work, finances, caregiving, or life events lasting >1 month.
    - *Include:*"high work stress", "financial worries", "caregiver burnout", "anxious".
    - *Exclude:*"stressed about exam next week" (acute).
- **Sleep Disturbance:** Insomnia, obstructive sleep apnea, or insufficient sleep duration.

    - *Include:*"can't fall asleep", "snores loudly", "sleeps 4 hours/night".
    - *Exclude:*"occasional bad dream".

4.4 Medication & Self-Management Adherence

Targets the "Pharmacological Control" pathway.

- **Non-Adherence:** Behavior indicating failure to follow prescribed treatment.
    - *Include:*"skips medication", "ran out of pills", "doesn't check sugar", "forgets insulin".
    - *Exclude:*"takes meds as prescribed", "checks sugar daily".

4.5 Clinical History & Symptoms

Targets "Disease Progression" markers.

- **Family History:** First-degree relatives (parents, siblings) with T2DM.
    - *Include:*"dad has diabetes", "mother on insulin", "family hx DM".
    - *Exclude:*"grandmother had diabetes" (unless specified as first-degree).
- **Polycystic Ovary Syndrome (PCOS):** Documented diagnosis in female patients.
- **Specific Symptoms:** Presence of classic T2DM symptoms (polyuria, polydipsia, unexplained weight loss).

5. Annotation Rules & Edge Cases

5.1 Negation Handling (CRITICAL)

All negations must be strictly observed. Use the **NegEx algorithm** logic.

- **Rule:** If a negation cue appears within 5 words before the risk factor, mark as **Absent (0)**.
- **Examples:**
    - "**Denies** fast food" → **Absent**.
    - "**No** history of diabetes in family" → **Absent**.
    - "Used to smoke, **quit** 5 years ago" → **Absent** (historical).

5.2 Temporal Context

Only annotate **Current** or **Chronic** status.

- **Current:** "Currently stressed", "still smokes".
- **Historical:** "Used to be sedentary", "smoked in college" → Mark as **Absent** unless specified otherwise.

5.3 Ambiguity & Uncertainty

- If the text is ambiguous (e.g., "maybe eats poorly"), mark as **Uncertain (?)**.
- If conflicting information exists (e.g., "exercises daily" vs. "mostly sits"), prioritize the **most recent** or **most severe** statement.

5.4 Abbreviation Expansion

Standardize common abbreviations before annotation:

- DM→ Diabetes Mellitus
- HTN→ Hypertension
- FHx→ Family History
- OB→ Obesity
- SOB→ Shortness of Breath

6. Annotation Interface & Output Format

Annotators used a custom web interface. The output was structured as a JSON object per record.

{
  "record_id": "6789",
  "text_source": "lifestyle_notes",
  "annotations": {
    "poor_diet_high_sugar": 1,
    "poor_diet_fast_food": 1,
    "physical_inactivity_sedentary_job": 1,
    "physical_inactivity_leisure": 0,
    "psychological_stress": 1,
    "sleep_disturbance": 0,

"med_adherence": 0,
"family_history": 1,
"pcos": 0,
"symptoms_polyuria": 1
},
"annotator_id": "PH_Researcher_02",
"confidence_score": 0.95,
"notes": "Clear indication of high stress and poor diet. Sedentary job mentioned."
}

7. Quality Assurance & Inter-Annotator Agreement (IAA)

7.1 IAA Metric

Fleiss' Kappa was calculated for the 300-record set.

- **Overall Kappa:** 0.81 (95% CI: 0.76–0.86) → **Almost Perfect Agreement**.

7.2 Disagreement Resolution Protocol

1. **Initial Pass:** Independent annotation.
2. **Adjudication:** All 3 annotators reviewed records where Kappa < 0.7.
3. **Final Consensus:** Majority vote determined the final label. If a tie occurred, the Endocrinologist's label was the tie-breaker.

8. Detailed Annotation Examples

**Text Excerpt**	**Correct Annotation**	**Rationale**
"Pt c/o fatigue, thirsty all the time. Drinks soda w/ every meal. Dad has DM. Works IT	poor_diet_high_sugar:1, family_history:1, physical_inactivity_sedentary_job:1, symptoms_polyuria:1	Identifies sugar intake, family history, sedentary work, and thirst symptom.

Text Excerpt	Correct Annotation	Rationale
job."		
"Denies smoking. Exercises 3x/wk. Stressful job but manages w/ yoga. Sleeps well."	med_adherence:0, psychological_stress:0	Negation ("Denies") and effective management ("yoga") override stress mention.
"Wt gain despite diet. Fast food 5x/wk. Mom & brother DM. Skips metformin often."	poor_diet_fast_food:1, family_history:1, med_adherence:0	Captures fast food, strong family history, and non-adherence.
"History of PCOS. Trying to lose weight. No regular exercise. Anxious about work."	pcos:1, physical_inactivity_leisure:1, psychological_stress:1	Correctly identifies PCOS, inactivity, and stress.

9. Ethical & Privacy Compliance

- **De-identification:** All Protected Health Information (PHI) was removed prior to annotation.
- **Data Security:** Annotators accessed data via secure, encrypted virtual machines with no internet access.
- **IRB Approval:** Annotation protocol approved under IRB waiver (see Section 9 of Main Manuscript).

Contact: For questions regarding this guideline, contact 1339732610@qq.com(Technical Lead: Cecilia).
