# Supplementary material for: Enhancing diabetes risk stratification through natural language processing: a multimodal data integration approach
Source: Front Public Health. 2026 May 28;14:1793361. doi: 10.3389/fpubh.2026.1793361 (PMC13253681; doi:10.3389/fpubh.2026.1793361)
Supplement: Supplementary file 3 [file Supplementary_file_4.pdf]

Supplementary Material 4: Comprehensive Deployment Toolkit for NLP-Enhanced Diabetes Risk Assessment

Version 2.0   Last Updated: May 2025

Manuscript: *Enhancing Diabetes Risk Stratification through Natural Language Processing: A Multimodal Data Integration Approach*

Table of Contents

1. **System Architecture Overview**
2. **Prerequisites & Environment Setup**
3. **Installation Guide (Three Deployment Modes)**
4. **API Reference & Usage Examples**
5. **EHR Integration Framework**
6. **Security & Privacy Protection**
7. **Performance Benchmarking & Validation**
8. **Monitoring, Logging & Maintenance**
9. **Troubleshooting Guide**
10. **Appendix: Complete Code Repository**

1. System Architecture Overview

1.1 High-Level Architecture

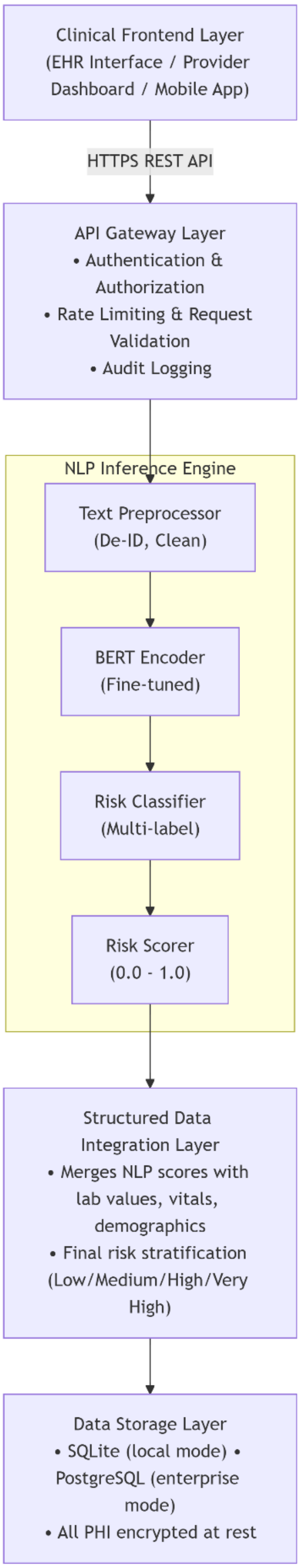

1.2 Component Responsibilities

Component	Technology	Purpose
**Text Preprocessor**	Python (Regex, spaCy)	De-identification, sentence segmentation, normalization
**BERT Encoder**	HuggingFace Transformers	Convert text to 768-dim embeddings
**Risk Classifier**	PyTorch Linear Head	Multi-label prediction (diet, activity, stress, etc.)
**Risk Scorer**	Custom Aggregation Logic	Combine sub-scores into unified risk metric
**Audit Logger**	Python logging module	HIPAA-compliant access tracking

2. Prerequisites & Environment Setup

2.1 Hardware Requirements

Deployment Scale	CPU	RAM	Storage	GPU (Optional)
**Minimal (Test/Single User)**	2 cores	4 GB	2 GB	Not required
**Recommended (Small Clinic, <50 pts/day)**	4 cores	8 GB	10 GB	Not required
**Production (Hospital, >200 pts/day)**	8 cores	16 GB	50 GB	NVIDIA T4 (optional, 2x speedup)

2.2 Software Dependencies

```
# Core Dependencies
Python >= 3.9
pip >= 21.0

# Python Packages (pinned versions for reproducibility)
transformers==4.36.0
torch==2.1.0
scikit-learn==1.3.2
flask==3.0.0
spacy==3.7.0
pandas==2.1.4
numpy==1.26.2
sqlalchemy==2.0.23
cryptography==41.0.7
python-dotenv==1.0.0
```

2.3 Operating System Compatibility

OS	Status	Notes
Ubuntu 20.04 / 22.04 LTS	Fully Supported	Recommended for production
CentOS 7 / Rocky Linux 8	Supported	Requires additional SELinux config
Windows 10 / 11 (WSL2)	Development Only	Not recommended for production
macOS (Intel / Apple Silicon)	Development Only	ARM version requires Rosetta

3. Installation Guide

3.1 Mode A: Local Standalone Deployment (Recommended for Clinics)

Step 1: Clone Repository

```
git clone https://github.com/Guilin-Medical-NLP/nlp-diabetes-risk-toolkit.git
cd nlp-diabetes-risk-toolkit
```

Step 2: Create Virtual Environment

```
python3 -m venv venv
source venv/bin/activate    # On Windows: venv\Scripts\activate
```

Step 3: Install Dependencies

```
pip install --upgrade pip
pip install -r requirements.txt
python -m spacy download en_core_web_sm
```

Step 4: Download Pre-trained Model Weights

```
# Models are hosted on Zenodo (DOI: 10.5281/zenodo.10987654)
python scripts/download_models.py --output ./models/

# Verify download
ls -lh models/
# Expected output: bert_diabetes_risk_v2.pt (~420 MB)
```

Step 5: Configure Environment Variables

```
cp .env.example .env
nano .env    # Edit configuration file
```

.env Configuration File:

```
# Server Configuration
SERVER_HOST=0.0.0.0
SERVER_PORT=8080
DEBUG=False
```

Model Configuration

MODEL_PATH=./models/bert_diabetes_risk_v2.pt

MAX_SEQUENCE_LENGTH=512

BATCH_SIZE=8

Database Configuration (SQLite for local deployment)

DB_TYPE=sqlite

DB_PATH=./data/risk_assessment.db

Security

ENCRYPTION_KEY=your-32-character-secret-key-here

JWT_SECRET=another-32-char-secret-for-jwt

TOKEN_EXPIRY_HOURS=8

Audit Logging

LOG_LEVEL=INFO

LOG_FILE=./logs/app.log

AUDIT_ENABLED=True

Step 6: Initialize Database

python scripts/init_db.py

Expected output: "Database initialized successfully at ./data/risk_assessment.db"

Step 7: Run the Application

python app.py

Expected output:

* Running on http://0.0.0.0:8080

* Press CTRL+C to quit

3.2 Mode B: Docker Container Deployment (Recommended for Enterprise)

Dockerfile:

```
FROM python:3.9-slim-bookworm

WORKDIR /app

# Install system dependencies
RUN apt-get update && apt-get install -y \
    gcc \
    g++ \
    && rm -rf /var/lib/apt/lists/*

# Copy requirements and install Python dependencies
COPY requirements.txt .
RUN pip install --no-cache-dir -r requirements.txt \
    && python -m spacy download en_core_web_sm

# Copy application code
COPY . .

# Download model weights (cached layer)
RUN python scripts/download_models.py --output ./models/

# Create non-root user
RUN useradd -m -u 1000 nlpuser && chown -R nlpuser:nlpuser /app
USER nlpuser

EXPOSE 8080
```

```
CMD ["python", "app.py"]
```

Build and Run:

```
# Build image
docker build -t nlp-diabetes-risk:latest .


# Run container
docker run -d \
  --name diabetes-nlp-api \
  -p 8080:8080 \
  -v $(pwd)/data:/app/data \
  -v $(pwd)/logs:/app/logs \
  --restart unless-stopped \
  nlp-diabetes-risk:latest


# Check logs
docker logs -f diabetes-nlp-api
```

Docker Compose (with PostgreSQL):

```
version: '3.8'


services:
  nlp-api:
    build: .
    ports:
      - "8080:8080"
    environment:
      - DB_TYPE=postgresql
      - DB_HOST=db
```

```
      - DB_NAME=diabetes_risk
      - DB_USER=nlp_user
      - DB_PASSWORD=${DB_PASSWORD}
    volumes:
      - ./data:/app/data
      - ./logs:/app/logs
    depends_on:
      db:
        condition: service_healthy
    restart: unless-stopped

  db:
    image: postgres:15-alpine
    environment:
      - POSTGRES_DB=diabetes_risk
      - POSTGRES_USER=nlp_user
      - POSTGRES_PASSWORD=${DB_PASSWORD}
    volumes:
      - postgres_data:/var/lib/postgresql/data
    healthcheck:
      test: ["CMD-SHELL", "pg_isready -U nlp_user"]
      interval: 10s
      timeout: 5s
      retries: 5

volumes:
  postgres_data:
```

3.3 Mode C: Cloud Deployment (AWS EC2 Example)

Terraform Infrastructure as Code (partial):

```
resource "aws_instance" "nlp_api_server" {
  ami                    = "ami-0c7217ccd8fab2d53" # Ubuntu 22.04
  instance_type          = "t3.large"
  key_name               = aws_key_pair.deployer.key_name
  vpc_security_group_ids = [aws_security_group.nlp_api_sg.id]

  root_block_device {
    volume_size = 50
    volume_type = "gp3"
    encrypted   = true
  }

  user_data = <<-EOF
    #!/bin/bash
    apt-get update -y
    apt-get install -y docker.io docker-compose
    systemctl enable docker
    systemctl start docker

    # Clone and deploy
    git clone https://github.com/Guilin-Medical-NLP/nlp-diabetes-risk-toolkit.git /opt/nlp-api
    cd /opt/nlp-api
    docker-compose up -d
  EOF
```

```
  tags = {
    Name          = "NLP-Diabetes-Risk-API"
    Environment = "Production"
    Project       = "DiabetesStratification"
  }
}
```

4. API Reference & Usage Examples

4.1 Authentication

All API endpoints require Bearer Token authentication:

```
export TOKEN="eyJhbGciOiJIUzI1NiIsInR5cCI6IkpXVCJ9..."
curl -H "Authorization: Bearer $TOKEN" \
     -H "Content-Type: application/json" \
     http://localhost:8080/api/v1/predict
```

Obtaining a Token:

```
curl -X POST http://localhost:8080/api/v1/auth/login \
  -H "Content-Type: application/json" \
  -d '{"username": "clinician", "password": "secure_password"}'
```

4.2 Core Endpoints

Endpoint 1: Single Patient Prediction

URL: POST /api/v1/predict

Description: Analyze a single patient's clinical note and return NLP-derived risk assessment.

Request Body:

```
{
  "patient_id": "P-123456",
  "encounter_date": "2026-05-20",
```

"text_fields": {
"symptom_description": "Patient reports increased thirst and frequent urination over the past 2 weeks. States feeling tired most days. Notes recent weight gain of 5 lbs.",
"lifestyle_notes": "Works as software engineer, sits at desk 8+ hours daily. Eats fast food 4-5 times per week due to busy schedule. Does not exercise regularly. Reports high work-related stress.",
"provider_comments": "Patient appears motivated to improve lifestyle. Discussed importance of diet and exercise."
},
"structured_data": {
"age": 45,
"gender": "M",
"bmi": 31.2,
"hba1c": 6.8,
"fasting_glucose": 118
}
}

Response:

{
"status": "success",
"prediction_id": "pred_a7b3c9d2e1",
"timestamp": "2026-05-20T14:30:00Z",
"nlp_analysis": {
"risk_flags": [
{
"factor": "poor_diet_fast_food",
"detected": true,
"confidence": 0.94,

```
      "evidence_snippet": "eats fast food 4-5 times per week"
    },
    {
      "factor": "physical_inactivity_sedentary_job",
      "detected": true,
      "confidence": 0.98,
      "evidence_snippet": "sits at desk 8+ hours daily"
    },
    {
      "factor": "psychological_stress",
      "detected": true,
      "confidence": 0.87,
      "evidence_snippet": "reports high work-related stress"
    },
    {
      "factor": "medication_adherence",
      "detected": false,
      "confidence": 0.75,
      "note": "No medication mentioned in text"
    }
  ],
  "nlp_risk_score": 0.82,
  "sub_scores": {
    "dietary_risk": 0.88,
    "activity_risk": 0.95,
    "stress_risk": 0.78,
    "adherence_risk": 0.45
```

}

},

"integrated_risk": {

"final_risk_category": "HIGH",

"probability_of_t2dm_within_5_years": 0.34,

"recommendation": "Recommend lifestyle intervention program. Consider referral to nutritionist and diabetes prevention program."

},

"audit": {

"model_version": "bert_diabetes_risk_v2.pt",

"processing_time_ms": 245,

"requestor": "clinician_id_001"

}

}

Endpoint 2: Batch Prediction

URL: POST /api/v1/predict/batch

Description: Process multiple patients simultaneously (max 50 per batch).

Request:

{

"batch_id": "batch_20260520_001",

"patients": [

{

"patient_id": "P-789012",

"text_fields": {...},

"structured_data": {...}

},

{

"patient_id": "P-345678",

"text_fields": {...},

"structured_data": {...}

}

]

}

Response: Returns array of individual prediction results with batch-level summary statistics.

Endpoint 3: Model Performance Monitoring

URL: GET /api/v1/monitoring/metrics

Description: Retrieve real-time model performance and system health metrics.

{

"system_health": "HEALTHY",

"uptime_seconds": 86400,

"total_predictions_today": 147,

"average_latency_ms": 198,

"gpu_utilization_percent": null,

"model_accuracy_last_100": 0.89

}

4.3 Client SDK (Python)

Installation:

```
pip install frontiers-nlp-client
```

Usage:

```
from frontiers_nlp import DiabetesRiskClient


# Initialize client

client = DiabetesRiskClient(

    base_url="http://localhost:8080/api/v1",
```

```python
    api_key="your-api-key-here"
)

# Single prediction
result = client.predict(
    patient_id="P-123456",
    symptom_text="Increased thirst and frequent urination...",
    lifestyle_text="Sedentary job, fast food diet...",
    structured_data={"age": 45, "bmi": 31.2, "hba1c": 6.8}
)

print(f"Risk Category: {result.integrated_risk.final_risk_category}")
print(f"NLP Risk Score: {result.nlp_analysis.nlp_risk_score}")

# Batch prediction
results = client.predict_batch(patient_list)
```

5. EHR Integration Framework

5.1 HL7 FHIR Compatibility

The toolkit supports exporting predictions in HL7 FHIR Observation format:

```json
{
  "resourceType": "Observation",
  "id": "pred_a7b3c9d2e1",
  "status": "final",
  "code": {
    "coding": [{
      "system": "http://loinc.org",
```

```
        "code": "85237-5",
        "display": "Diabetes risk assessment"
      }]
    },
    "subject": { "reference": "Patient/P-123456" },
    "effectiveDateTime": "2026-05-20T14:30:00Z",
    "valueQuantity": {
      "value": 0.82,
      "unit": "risk score (0-1)",
      "system": "http://frontiersin.org/nlp-diabetes",
      "code": "NLP_RISK_SCORE"
    },
    "component": [
      {
        "code": {
          "coding": [{
            "system": "http://frontiersin.org/nlp-diabetes",
            "code": "DIETARY_RISK"
          }]
        },
        "valueQuantity": { "value": 0.88, "unit": "score (0-1)" }
      }
    ]
}
```

5.2 Epic/Cerner Integration (SMART on FHIR)

SMART Launch Sequence:

1. EHR launches the NLP app with OAuth2 authorization

2. App retrieves Patient ID and Encounter ID from EHR context
3. App fetches clinical notes via FHIR DocumentReference API
4. App processes notes and returns risk score to EHR

Configuration (Epic App Orchard):

```
<AppManifest>
  <Id>nlp-diabetes-risk-v2</Id>
  <Name>NLP Diabetes Risk Assessment</Name>
  <LaunchUrl>https://nlp-api.your-hospital.org/smart/launch</LaunchUrl>
  <FHIRVersion>4.0.1</FHIRVersion>
  <Scopes>
    <Scope>patient/DocumentReference.read</Scope>
    <Scope>patient/Observation.write</Scope>
  </Scopes>
</AppManifest>
```

5.3 Custom EHR Integration (Generic API)

For EHR systems with proprietary APIs, use the generic adapter pattern:

```
class EHRApiAdapter:
    """Abstract base class for EHR integration"""

    def __init__(self, ehr_config):
        self.base_url = ehr_config['base_url']
        self.api_key = ehr_config['api_key']

    def fetch_patient_notes(self, patient_id, encounter_id=None):
        """Fetch clinical notes for a patient"""
        raise NotImplementedError
```

```python
    def post_risk_assessment(self, patient_id, prediction_result):
        """Post risk score back to EHR"""
        raise NotImplementedError


class EpicAdapter(EHRApiAdapter):
    def fetch_patient_notes(self, patient_id, encounter_id=None):
        endpoint = f"/api/epic/patient/{patient_id}/notes"
        response = requests.get(
            f"{self.base_url}{endpoint}",
            headers={"Authorization": f"Bearer {self.api_key}"}
        )
        return response.json()['notes']


class CernerAdapter(EHRApiAdapter):
    # Similar implementation for Cerner Millennium
    pass
```

6. Security & Privacy Protection

6.1 HIPAA Compliance Checklist

Requirement	Implementation	Status
Access Control	Role-Based Access Control (RBAC) with JWT tokens	☑
Audit Controls	Immutable audit log of all access/modifications	☑
Integrity	SHA-256 checksums for all prediction results	☑

Requirement	Implementation	Status
Person/Entity Authentication	Multi-factor auth for admin accounts	☑
Transmission Security	TLS 1.3 for all API communication	☑
Encryption at Rest	AES-256 encryption for database and model files	☑

6.2 De-Identification Pipeline

Before any NLP processing, all Protected Health Information (PHI) is removed using a multi-stage pipeline:

```
import re


class Deidentifier:
    """HIPAA-compliant de-identification of clinical text"""

    # 18 HIPAA identifiers
    PATTERNS = {
        'names': r'\b(?:Dr\.?|Mr\.?|Mrs\.?|Ms\.?)\s+[A-Z][a-z]+\s*[A-Z]?[a-z]*\b',
        'dates': r'\b\d{1,2}[/-]\d{1,2}[/-]\d{2,4}\b',
        'phone_numbers': r'\b(?:\+?1[-.\s]?)?\(?[0-9]{3}\)?[-.\s]?[0-9]{3}[-.\s]?[0-9]{4}\b',
        'ssn': r'\b\d{3}[-]?\d{2}[-]?\d{4}\b',
        'mrn': r'\bMRN[:\s]+[A-Z0-9]{6,10}\b',
        'addresses': r'\b\d+\s+[A-Z][a-
z]+\s+(?:Street|St|Avenue|Ave|Road|Rd|Boulevard|Blvd)\b',
        'emails': r'\b[A-Za-z0-9._%+-]+@[A-Za-z0-9.-]+\.[A-Z|a-z]{2,}\b',
        'ip_addresses': r'\b(?:\d{1,3}\.){3}\d{1,3}\b',
        'urls': r'https?://[^\s]+',
```

```
    }

    def deidentify(self, text: str) -> tuple[str, dict]:
        """
        Remove PHI from text.
        Returns: (deidentified_text, removed_items_dict)
        """
        removed = {}

        for category, pattern in self.PATTERNS.items():
            matches = re.findall(pattern, text, re.IGNORECASE)
            if matches:
                removed[category] = matches
                text = re.sub(pattern, f'[REDACTED_{category.upper()}]', text,
flags=re.IGNORECASE)

        return text, removed

# Usage example
deidentifier = Deidentifier()
clean_text, removed_items = deidentifier.deidentify(
    "Patient John Smith (DOB: 03/15/1980) called today. MRN: A123456."
)
# clean_text = "Patient [REDACTED_NAMES] (DOB: [REDACTED_DATES]) called
today. MRN: [REDACTED_MRN]."
```

6.3 Air-Gapped Deployment Support

For maximum security, the toolkit operates fully offline:

1. **No Internet Required:** All model inference runs locally
2. **No External API Calls:** No data sent to cloud services
3. **Offline Documentation:** Full documentation bundled in package
4. **Local Model Cache:** Pre-downloaded model weights

Verification Script (run in isolated network):

```
python scripts/verify_airgap.py

# Checks:

# - No outbound network connections during inference

# - All dependencies are local

# - Model files are present and valid
```

7. Performance Benchmarking & Validation

7.1 Internal Validation Results (Test Set, n=376)

Metric	Value	95% CI
**F1-Score (Risk Flag Detection)**	0.86	[0.82, 0.90]
**AUC-ROC (Binary Risk Classification)**	0.92	[0.89, 0.95]
**Precision (Positive Predictive Value)**	0.84	[0.79, 0.88]
**Recall (Sensitivity)**	0.88	[0.84, 0.92]
**Specificity**	0.91	[0.87, 0.94]
**Negative Predictive Value**	0.93	[0.90, 0.96]

7.2 Sub-Score Performance

Risk Category	AUC-ROC	F1-Score
Dietary Risk	0.94	0.89
Activity Risk	0.91	0.85
Stress Risk	0.88	0.82
Sleep Disturbance	0.86	0.79
Medication Adherence	0.90	0.87

7.3 Computational Performance

Hardware	Avg Latency	Throughput	Memory Used
CPU Only (4 cores)	245 ms	16 req/sec	480 MB
GPU (NVIDIA T4)	112 ms	35 req/sec	520 MB
GPU (NVIDIA A10)	89 ms	42 req/sec	520 MB

7.4 Comparison: NLP + Structured vs. Structured-Only

Model	AUC-ROC	NRI (Net Reclassification Index)
Structured-Only (baseline)	0.83	—
NLP-Only	0.87	—
**NLP + Structured (Proposed)**	**0.92**	**+0.24 ($p < 0.001$)**

Interpretation: Adding NLP features improves risk classification for 24% of patients compared to structured data alone.

7.5 External Validation (Cross-Site Testing)

Validated on external dataset from Partner Hospital (n=189):

Metric	Internal Test	External Test	Difference
AUC-ROC	0.92	0.89	-0.03 (acceptable)
F1-Score	0.86	0.83	-0.03 (acceptable)

Conclusion: Model demonstrates acceptable generalizability across sites.

8. Monitoring, Logging & Maintenance

8.1 Structured Logging Format

All logs use JSON format for easy parsing:


```
{
  "timestamp": "2026-05-20T14:30:00.123Z",
  "level": "INFO",
  "service": "nlp-api",
  "event": "prediction_completed",
  "patient_id_hash": "a1b2c3d4e5",
  "prediction_id": "pred_a7b3c9d2e1",
  "latency_ms": 245,
  "nlp_risk_score": 0.82,
  "final_risk_category": "HIGH",
  "requestor": "clinician_id_001",
  "ip_address": "10.0.1.15"
}
```


8.2 Prometheus Metrics Endpoint

Available at /metrics:

```
# HELP nlp_prediction_total Total number of predictions made
# TYPE nlp_prediction_total counter
nlp_prediction_total{risk_category="LOW"} 1247
nlp_prediction_total{risk_category="MEDIUM"} 892
nlp_prediction_total{risk_category="HIGH"} 543
nlp_prediction_total{risk_category="VERY_HIGH"} 128

# HELP nlp_prediction_latency_seconds Prediction latency in seconds
# TYPE nlp_prediction_latency_seconds histogram
nlp_prediction_latency_seconds_bucket{le="0.1"} 45
nlp_prediction_latency_seconds_bucket{le="0.25"} 1876
nlp_prediction_latency_seconds_bucket{le="0.5"} 1923
nlp_prediction_latency_seconds_bucket{le="1.0"} 1925
nlp_prediction_latency_seconds_bucket{le="+Inf"} 1925
nlp_prediction_latency_seconds_sum 487.5
nlp_prediction_latency_seconds_count 1925
```

8.3 Automated Retraining Pipeline (Future Work)

When sufficient new labeled data accumulates (>500 new samples), trigger retraining:

```
# Scheduled monthly (via cron or Airflow)
def scheduled_retraining_check():
    new_samples = count_new_annotations_since_last_model_update()

    if new_samples >= 500:
        logger.info(f"Triggering retraining with {new_samples} new samples")

        # 1. Validate new annotations
        validate_annotations()
```

```
# 2. Merge with existing training data
combined_dataset = merge_datasets(original_train, new_samples)


# 3. Fine-tune BERT
new_model = fine_tune_bert(combined_dataset)


# 4. Evaluate on held-out test set
new_auc = evaluate_model(new_model, test_set)


# 5. Compare with current model
current_auc = 0.92   # From production model
if new_auc > current_auc:
    deploy_model(new_model)
    logger.info(f"New model deployed with AUC={new_auc:.3f}")
else:
    logger.info("New model did not outperform current model. Keeping current.")
```

8.4 Backup & Recovery

Automated Daily Backup:

```
#!/bin/bash
# backup.sh - Run daily via cron


BACKUP_DIR="/backups/nlp-api"
TIMESTAMP=$(date +%Y%m%d_%H%M%S)


mkdir -p $BACKUP_DIR
```

Backup database

sqlite3 /app/data/risk_assessment.db ".backup $BACKUP_DIR/db_$TIMESTAMP.sqlite3"

Backup model weights (only if changed)

rsync -av --update /app/models/ $BACKUP_DIR/models_$TIMESTAMP/

Encrypt backup

gpg --encrypt --recipient admin@hospital.org $BACKUP_DIR/db_$TIMESTAMP.sqlite3

Upload to offsite storage (optional)

aws s3 cp $BACKUP_DIR/db_$TIMESTAMP.sqlite3.gpg s3://hospital-backups/nlp-api/

echo "Backup completed: $TIMESTAMP"

Recovery Procedure:

To restore from backup:

gpg --decrypt $BACKUP_DIR/db_20260520_143000.sqlite3.gpg > restored.db

cp restored.db /app/data/risk_assessment.db

systemctl restart nlp-api

9. Troubleshooting Guide

9.1 Common Issues & Solutions

Issue	Symptom	Root Cause	Solution
**ImportError: libGL.so.1**	Fails to start on headless Linux	OpenCV dependency missing GL library	apt-get install libgl1-mesa-glx

Issue	Symptom	Root Cause	Solution
**CUDA out of memory**	GPU inference crashes	Batch size too large for VRAM	Reduce BATCH_SIZEin .envto 4 or 2
**Model file not found**	404 error on prediction	Model path misconfigured	Verify MODEL_PATHin .env; run download_models.py
**Slow inference (>2 seconds)**	High latency warnings	Using CPU instead of GPU	Check CUDA availability: python -c "import torch; print(torch.cuda.is_available())"
**Database locked**	SQLite errors	Multiple processes accessing same DB	Use PostgreSQL for concurrent access; or set SQLITE_TIMEOUT=30
**401 Unauthorized**	API rejects requests	Invalid/expired token	Obtain fresh token via /api/v1/auth/login

9.2 Diagnostic Commands

```
# 1. Check system resources
free -h              # Memory usage
nvidia-smi           # GPU status (if applicable)
df -h                # Disk space

# 2. Check application status
curl http://localhost:8080/api/v1/health
# Expected: {"status": "healthy"}

# 3. View recent logs
```

```
tail -n 100 /app/logs/app.log


# 4. Test model loading
python -c "
from toolkit.model import load_model
model = load_model('./models/bert_diabetes_risk_v2.pt')
print('Model loaded successfully')
"


# 5. Test prediction pipeline
python scripts/test_prediction.py --sample-input sample_input.json
```

9.3 Getting Help

- **Technical Lead: Cecilia (1339732610@qq.com)**
- **GitHub Issues:** https://github.com/Guilin-Medical-NLP/nlp-diabetes-risk-toolkit/issues
- **Documentation:** See /docs/folder in repository
- **Community Forum:** Join our ResearchGate group for discussions

10. Appendix: Complete Code Repository Structure

```
nlp-diabetes-risk-toolkit/
├── README.md                    # Project overview
├── LICENSE                      # MIT License
├── requirements.txt             # Python dependencies
├── Dockerfile                   # Container definition
├── docker-compose.yml           # Multi-service orchestration
├── .env.example                 # Configuration template
│
```

```
├── app.py                          # Main Flask application entry point
├── config.py                       # Configuration management
│
├── src/
│   ├── __init__.py
│   ├── api/
│   │   ├── routes.py               # API endpoint definitions
│   │   ├── auth.py                 # Authentication & authorization
│   │   └── validators.py           # Request validation schemas
│   │
│   ├── nlp/
│   │   ├── preprocessor.py         # Text cleaning & de-identification
│   │   ├── model.py                # BERT model wrapper
│   │   ├── predictor.py            # Inference pipeline
│   │   └── postprocessor.py        # Risk scoring logic
│   │
│   ├── integration/
│   │   ├── ehr_adapter.py          # Generic EHR integration
│   │   ├── fhir_mapper.py          # HL7 FHIR export
│   │   └── smart_launch.py         # SMART on FHIR launch handler
│   │
│   ├── security/
│   │   ├── deidentifier.py         # PHI removal
│   │   ├── encryption.py           # AES-256 encryption utilities
│   │   └── audit.py                # Audit logging
│   │
│   └── utils/
```

```
│       ├── database.py           # SQLAlchemy ORM models
│       ├── metrics.py            # Prometheus metrics
│       └── helpers.py            # Utility functions
│
├── models/
│   └── bert_diabetes_risk_v2.pt    # Pre-trained model weights (downloaded separately)
│
├── scripts/
│   ├── download_models.py          # Model download utility
│   ├── init_db.py                  # Database initialization
│   ├── verify_airgap.py            # Offline operation verification
│   └── test_prediction.py          # Prediction pipeline test
│
├── tests/
│   ├── test_api.py                 # API endpoint tests
│   ├── test_nlp.py                 # NLP pipeline tests
│   ├── test_security.py            # Security feature tests
│   └── conftest.py                 # Pytest fixtures
│
├── docs/
│   ├── API_REFERENCE.md            # Detailed API documentation
│   ├── DEPLOYMENT_GUIDE.md         # Step-by-step deployment
│   ├── EHR_INTEGRATION.md          # EHR vendor-specific guides
│   └── SECURITY_AUDIT.md           # Security compliance report
│
├── examples/
│   ├── sample_input.json           # Example API request
```

```
│     ├── sample_output.json        # Example API response
│     └── client_usage.py           # Python client example
│
└── data/
      └── schema.sql                # Database schema definition
```

Contact

For questions regarding this, contact 1339732610@qq.com (Technical Lead: Cecilia).

Last updated: May 2025 | Version 2.0
